# Y-27632 acts beyond ROCK inhibition to maintain epidermal stem-like cells in culture

**DOI:** 10.1242/jcs.260990

**Published:** 2023-09-12

**Authors:** Travis A. Witkowski, Bin Li, Jason G. Andersen, Bhavna Kumar, Edmund A. Mroz, James W. Rocco

**Affiliations:** ^1^ Department of Otolaryngology – Head and Neck Surgery, The Ohio State University Wexner Medical Center, Columbus, OH 43210, USA; ^2^ The James Cancer Hospital and Solove Research Institute, The Ohio State University, Columbus, OH 43210, USA; ^3^ The Ohio State University Comprehensive Cancer Center – James, The Ohio State University, Columbus, OH 43210, USA

**Keywords:** Conditional reprogramming, Epidermal stem cells, Rho-associated kinase, Rho-associated kinase inhibitors, Y-27632

## Abstract

Conditional reprogramming is a cell culture technique that effectively immortalizes epithelial cells with normal genotypes by renewing epidermal stem cells. Y-27632, a compound that promotes conditional reprogramming through an unknown mechanism, was developed to inhibit the two Rho-associated kinase (ROCK) isoforms. We used human foreskin keratinocytes (HFKs) to study the role of Y-27632 in conditional reprogramming and learn how ROCKs control epidermal stem cell renewal. In conditional reprogramming, Y-27632 increased HFK adherence to culture dishes, progression through S, G2 and M phases of the cell cycle, and epidermal stem cell marker levels. Although this correlated with ROCK inhibition by Y-27632, we generated CRISPR–Cas9-mediated HFK ROCK knockouts to test the direct role of ROCK inhibition. Knockout of single ROCK isoforms was insufficient to disrupt ROCK activity or promote HFK propagation without Y-27632. Although ROCK activity was reduced, HFKs with double knockout of ROCK1 and ROCK2 still required Y-27632 to propagate. Y-27632 was the most effective among the ROCK inhibitors we tested at promoting HFK proliferation and epidermal stem cell marker expression. Thus, the ability of Y-27632 to promote an epidermal stem cell state in conditional reprogramming not only depends upon ROCK inhibition but also acts via as-yet-unidentified mechanisms. Epidermal stem cell renewal might in part be regulated by ROCKs, but also involves additional pathways.

## Teaser

**Summary:** Y-27632 conditionally reprograms epidermal stem cells not only by inhibiting Rho kinases but also via an unidentified mechanism, contrary to the presumption that Rho kinase inhibition alone renews epidermal stem cells.

## INTRODUCTION

The conditional reprogramming cell culture technique provides replicative immortality, facilitating the *in vitro* propagation of both normal and diseased epithelial cells. Conditional reprogramming was initially developed as a method to culture normal epithelial cells, like keratinocytes, but has been expanded to other research areas (Chapman et al., 2014, 2010; Liu et al., 2012; Suprynowicz et al., 2012). Adding Y-27632, an inhibitor of Rho-associated protein kinases (ROCKs) to the Rheinwald–Green keratinocyte culture method reportedly allows epithelial cells to propagate indefinitely, unlike other techniques that only support a limited number of divisions (Chapman et al., 2010, 2014; Liu et al., 2012, 2017). Conditionally reprogrammed cultures express markers consistent with epidermal stem cell populations (Suprynowicz et al., 2012). Conditional reprogramming is so named because this reprogramming into epithelial stem-like cells is dependent upon regularly adding Y-27632 to cultures. When Y-27632 is removed, epithelial cultures undergo differentiation and stop dividing. As Y-27632 promotes epidermal stem cell renewal in conditionally reprogrammed cultures, we studied the role of Y-27632 in conditional reprogramming to identify how ROCKs control epidermal stem cell renewal.

Conditional reprogramming has been applied clinically, with its first clinical use to culture cells from benign but airway-obstructing recurrent respiratory papillomas (RRPs) along with non-diseased tissue from the same patient. Researchers have used conditional reprogramming to identify selective RRP-targeting compounds *in vitro* that cure RRPs in patients (Yuan et al., 2012). A similar culture method has been used in a gene therapy study to treat a blistering skin disease. Investigators were able to correct the laminin 332 mutation causing junctional epidermolysis bullosa in skin cells from the patient and then propagate the corrected cells into skin grafts for transplantation (Hirsch et al., 2017). Conditional reprogramming has been used in preclinical cystic fibrosis culture models (Zhang et al., 2018). Across cancer subtypes, conditional reprogramming is used to generate tumor cell line biobanks and to propagate normal and tumor cells from the same individual (Liu et al., 2017; Palechor-Ceron et al., 2019).

Beyond the investigational uses of conditional reprogramming to culture cells, conditional reprogramming also provides a unique model to study replicative immortality via maintaining epidermal stem cells. Epidermal stem cells are regulated by a variety of cell-intrinsic and -extrinsic signals, which remain incompletely characterized (Blanpain and Fuchs, 2009). Immortalization is a hallmark of tumorigenesis and is typically associated with irreversible somatic mutations that promote cancer (Hanahan and Weinberg, 2011). Y-27632 promotes epidermal stem cell marker expression and provides replicative immortality to epithelial cell cultures in a reversible, chemically dependent manner. Thus, understanding how Y-27632 promotes conditional reprogramming might provide insights into stem cell regulation pathways, in both normal tissue homeostasis and replicative immortality in cancers.

The specific role of Y-27632 in conditional reprogramming remains unclear. Y-27632 was developed to inhibit ROCKs (Ishizaki et al., 2000; Uehata et al., 1997). Investigators have hypothesized, but have not demonstrated, that conditional reprogramming depends on inhibition of ROCK kinase activity by Y-27632 (Liu et al., 2012; Roshan et al., 2016). The presumed role of Y-27632 in driving conditional reprogramming by inhibiting ROCKs has revealed two areas that require further investigation. The two ROCKs isoforms, ROCK1 and ROCK2, are both targeted by Y-27632 with similar *in vitro* binding affinities yet reportedly control keratinocyte differentiation with opposite isoform-dependent effects; however, the studies reporting these effects were conducted using immortalized cells lines that do not require Y-27632 to propagate (Lock and Hotchin, 2009). As separate roles have been attributed to the ROCK isoforms in keratinocyte differentiation, we aimed to test the specific contributions of each ROCK isoform in conditional reprogramming. Y-27632 also non-specifically inhibits other kinases besides the ROCKs *in vitro*, as do most ATP-competitive kinase inhibitors (Vigil et al., 2012). As Y-27632 can also inhibit other kinases besides ROCKs, we aimed to test the specificity of Y-27632 towards ROCKs in conditional reprogramming. Complementary genetic and pharmacological approaches have been used previously to resolve drug mechanisms of action (Gonçalves et al., 2020). We used CRISPR–Cas9-mediated knockouts and additional ROCK inhibitors to interrogate the role of ROCK inhibition in conditional reprogramming. Our results suggest that the ability of Y-27632 to conditionally reprogram keratinocytes depends upon activity beyond its known function as a ROCK inhibitor. Our studies show that Y-27632 promotes epidermal stem cell renewal not only by inhibiting ROCKs but also via unidentified mechanisms.

## RESULTS

### Y-27632 has multiple roles in promoting human foreskin keratinocyte culture

**Fig. 1.**
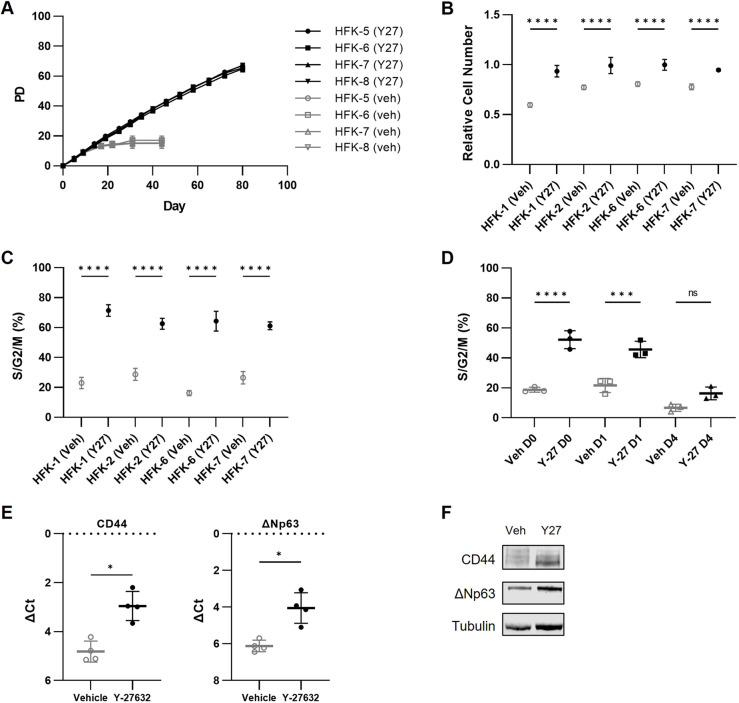
**Y-27632 has multiple roles in promoting HFK culture.** (A) Newly generated HFK strains were cultured in conditional reprogramming conditions for the first ten PDs. After ten PDs, early passage HFKs were either cultured in conditioned medium with 10 µM Y-27632 (Y27) or without Y-27632 (veh). Four from a total of eight HFK strains are plotted, with each strain cultured in technical triplicate. The data from this long-term experiment for the other four HFK strains can be found in Fig. S1C. Cell counts taken every 5 days were used to calculate the cumulative PDs of each culture, which were performed in triplicate and averaged across each treatment. (B) HFK strains were labeled with a nuclear-localized red fluorescent protein (nucRFP) to aid in automated cell counting, then plated either with or without 10 µM Y-27632 following trypsinization in six technical replicates per HFK strain. Viable, adherent HFKs were counted based on nucRFP signal every 3 h for 48 h after the HFKs were plated. The number of adherent HFKs after 24 h relative to the initial number of HFKs plated is plotted. Complete timecourses are plotted in Fig. S1E. Unpaired two-tailed Student's *t*-tests within each HFK strain comparing plating conditions with or without Y-27632 showed a significant decrease in cell number after 24 h without Y-27632 (****P<0.0001). (C) Four Fucci-labeled HFK strains were plated with Y-27632, then cultured with or without Y-27632. RFP- and GFP-expressing HFKs were counted with an Incucyte automated cell imager every 4 h, in six technical replicates per treatment. The fraction of HFKs in S/G2/M for four HFK strains at 24 h is plotted. Since the S/G2/M and G0/G1 fractions are the inverse of one another, we plotted only the fraction of HFKs in S/G2/M. Complete timecourses are plotted in Fig. S1F. Unpaired two-tailed Student's *t*-tests demonstrated a significant difference in S/G2/M fraction at 24 h between cultures with or without Y-27632 (****P<0.0001). (D) Fucci-labeled HFK strains were plated with Y-27632, then cultured with (Y-27 D0) or without Y-27632 (Veh D0). Additionally, Y-27632 (or vehicle) was re-added after either 1 day (D1) or 4 days (D4) without Y-27632, in treatments performed in technical triplicate. The graph shows the S/G2/M fraction 24 h after Y-27632 or vehicle was added back (or 24 h for D0), for one of four HFK strains (HFK-2; see Fig. S1G for timecourses of all strains). The 24 h time point was chosen to allow HFKs enough time to enter the cell cycle, given the timecourses identified in earlier experiments, as in Fig. 1C. Paired two-tailed Student's *t*-tests showed a significant increase in the fraction of HFKs in S/G2/M when Y-27632 was added back after 1 day without Y-27632 (Y-27 D1) relative to addition of vehicle (Veh D1). There was no significant difference when Y-27632 was added back after 4 days without Y-27632 (Y-27 D4) relative to addition of vehicle (Veh D4). ***P<0.001; ****P<0.0001; ns, not significant. (E) RT-qPCR was performed on cDNA from RNA harvested from four HFK strains cultured in standard conditional reprogramming conditions (Y-27632) or 5 days after Y-27632 removal (Vehicle). Each point represents the average across technical triplicates for each of the four HFK strains. GAPDH served as an internal control, and CD44 and ΔNp63 served as epidermal stem cell markers. Data are plotted as the difference between the threshold cycle for the marker gene and the internal GAPDH control (ΔCt). Paired two-tailed Student's *t*-tests showed a significant difference in the mRNA levels of these genes between vehicle and Y-27632 conditions (*P<0.05). (F) Western blots were performed on protein lysates from HFKs cultured in standard conditional reprogramming conditions (Y-27632) or 5 days after Y-27632 removal (Veh). One representative western blot from one of the two strains tested (HFK-1) is shown here. Tubulin served as an internal loading control, and CD44 and ΔNp63 served as epidermal stem cell markers. In A–E, data are presented as mean±s.d.

Before investigating the specific role of ROCKs in conditional reprogramming, we identified aspects of conditional reprogramming that depend on Y-27632. Neonatal human foreskin keratinocytes (HFKs) served as a model to study how Y-27632 conditionally reprograms epithelial cells. Lineage-specific marker expression studies confirmed that each of the eight HFK strains we isolated were keratinocytes (Fig. S1A,B). The HFK strains established in this study depended upon Y-27632 for conditional reprogramming, as reported previously (Chapman et al., 2014, 2010; Liu et al., 2012). Early passage conditionally reprogrammed HFK strains subsequently cultured without Y-27632 completely stopped dividing after five population doublings (PDs) following Y-27632 withdrawal, whereas the same strains continued to propagate in the presence of Y-27632 (Fig. 1A; Fig. S1C). Identifying pathways that could substitute for Y-27632 to promote HFK propagation could reveal how Y-27632 conditionally reprograms HFKs.

Conditional reprogramming enables serial subculturing of keratinocytes, and the cells must be passaged from one tissue culture dish to the next for extended propagation. We evaluated how Y-27632 could affect a single passage, as long-term culture over multiple passages requires repeated successes at each step of subculturing: enduring enzymatic digestion, surviving single-cell suspension, adhering to new culture dishes and then resuming cell division. Previous studies have found that Y-27632 enhances keratinocyte colony formation but does not block dissociation-induced apoptosis in keratinocytes (Terunuma et al., 2009). We thus evaluated the previously uninvestigated roles of Y-27632 in other aspects of passaging HFKs.

Y-27632 helped HFKs adhere to cell culture dishes, similar to the reported role of Y-27632 in human embryonic stem cell culture (Chen et al., 2010). During typical HFK culture, trypsinized HFKs are given 24 h to re-adhere, after which the HFKs divide. We hypothesized that Y-27632 promotes conditional reprogramming by allowing viable HFKs to re-adhere during subculture. To test this hypothesis, we trypsinized then replated equal numbers of HFKs either with or without Y-27632 and counted the viable HFKs over the following 48 h. Whereas the number of HFKs plated in Y-27632 remained consistent for the first 24 h, indicating that the HFKs had not yet divided, the number of HFKs plated without Y-27632 decreased by 20% over the first 24 h (Fig. 1B; Fig. S1E). Y-27632 thus prevented an initial decrease in HFKs during typical passaging conditions. Given these results, in all other experiments we plated HFKs with Y-27632 to control for adherence.

Y-27632 promoted cell cycle progression after we controlled for the ability of Y-27632 to promote HFK adherence. We hypothesized that, beyond promoting adherence, Y-27632 enhances HFK cell cycle progression. We used four independent HFK strains that were transduced with the fluorescent ubiquitylation-based cell cycle indicator (Fucci) system to monitor the cell cycle in live keratinocytes. With the Fucci system, red fluorescent protein (RFP) accumulates during G1 and G0 phases (G1/G0), whereas green fluorescent protein (GFP) accumulates during S, G2 and M phases (S/G2/M), allowing continuous monitoring of the cycle in individual cells (Sakaue-Sawano et al., 2008). Y-27632 treatment increased the fraction of HFKs in S/G2/M relative to that in vehicle-treated HFKs after 24 h, as evaluated using Incucyte live-cell imaging (Fig. 1C; Fig. S1F).

We extended studies with the Fucci system to assess whether Y-27632 is required beyond stimulation of the cell cycle. If Y-27632 simply promoted cell cycle progression, then HFKs would resume cell division whenever Y-27632 was added. If Y-27632 inhibits differentiation, as reported previously (Chapman et al., 2014), then HFKs would undergo irreversible cell differentiation after Y-27632 withdrawal and would not resume cell division upon re-addition of Y-27632. We removed Y-27632 from HFK cultures, then added back Y-27632 at different time intervals. When we added back Y-27632 to HFKs after 1 day without Y-27632, HFKs readily re-entered the cell cycle, as indicated by the increase in the fraction of HFKs in S/G2/M. However, when we added back Y-27632 to HFKs after 4 days without Y-27632, viable HFKs did not re-enter S/G2/M (Fig. 1D; Fig. S1G). Removal of Y-27632 had an additional, irreversible effect consistent with its reported role in inhibiting differentiation that developed in 1–4 days, within the typical duration of a single passage.

We hypothesized that HFKs did not re-enter S/G2/M after extended Y-27632 withdrawal because of a decrease in epidermal stem-like cells. Previous studies have reported that conditional reprogramming increases the levels of the epidermal stem cell markers ΔNp63 (encoded by *TP63*) and CD44, relative to other culture systems (Suprynowicz et al., 2012). We found that the increased ΔNp63 and CD44 levels seen during conditional reprogramming were Y-27632 dependent. We observed decreases in ΔNp63 and CD44 mRNA levels (Fig. 1E) and protein levels (Fig. 1F; Fig. S1D) at 5 days after Y-27632 removal. These studies identified the role of Y-27632 in conditional reprogramming and provided a series of simple assays to test the specific role of ROCKs in conditional reprogramming.

### Conditional reprograming conditions limit ROCK activity in HFKs

Although conditional reprogramming is presumed to be driven by inhibition of ROCKs by Y-27632, no study to date has shown that ROCK activity is specifically disrupted in conditionally reprogrammed cultures. All conditionally reprogrammed HFK strains expressed both ROCK1 and ROCK2 protein (Fig. S2A), the nominal targets of Y-27632. HFK strains also had similar ROCK1 and ROCK2 mRNA levels within each strain (Fig. S2B), which indicated that the HFK strains did not preferentially express a single ROCK isoform.

**Fig. 2.**
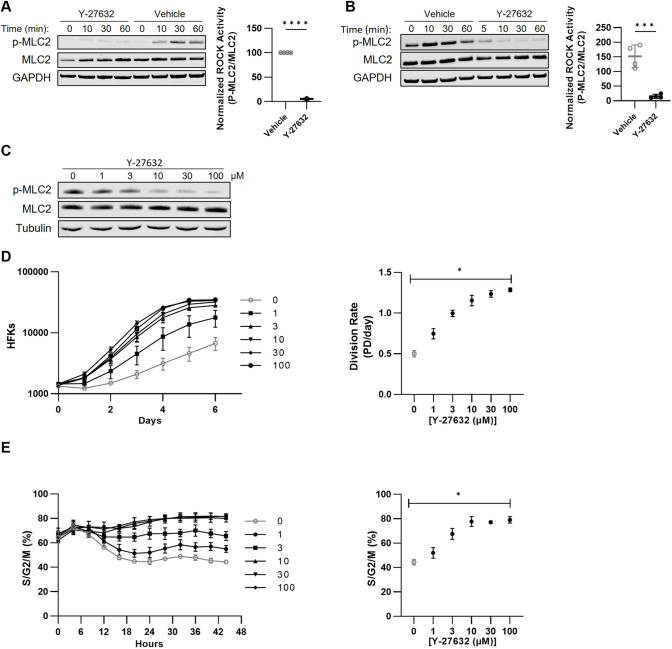
**Conditional reprogramming limits ROCK activity in HFKs.** (A) HFKs were cultured with conditioned medium containing 10 µM Y-27632. The medium was removed, HFKs were washed with PBS to remove any residual Y-27632, then conditioned medium containing Y-27632 or vehicle was added back. Protein lysates were harvested over the first hour following Y-27632 removal, across four HFK strains. Representative western blots from HFK-2 are shown here. The relative p-MLC2 signal normalized to MLC2 and GAPDH was quantified across the four strains. An unpaired two-tailed Student's *t*-test evaluated the difference in p-MLC2 signal between HFKs where Y-27632 was maintained or removed for 1 h, across all four strains (****P<0.0001). (B) To allow ROCK activity to return to its uninhibited level in HFKs, we washed HFKs with PBS, as described in A, then cultured the HFKs in conditioned medium without Y-27632 for 1 h. We added back 10 µM Y-27632 or vehicle then harvested protein lysates over the following 60 min. Western blots of lysates from HFK-2 are shown here and are representative of the four strains tested. The relative p-MLC2 signal, normalized to MLC2 and GAPDH, was quantified across the four strains 30 min after re-adding Y-27632 or vehicle. An unpaired two-tailed Student's *t*-test evaluated the differences in p-MLC2 signal between when Y-27632 was added back to HFKs or still removed, across all four strains (***P<0.001). (C) To allow ROCK activity to return to its uninhibited baseline level, we removed Y-27632 from conditionally reprogrammed HFKs, as described in A, for 24 h prior to adding back Y-27632. Protein lysates were harvested from two HFK strains, 24 h after adding Y-27632 at the indicated concentrations. The representative western blots from HFK-2 lysates show p-MLC2 and total MLC2 to evaluate ROCK activity, with tubulin shown as a loading control. (D) RFP-labeled HFKs were plated with 10 µM Y-27632, then washed with PBS and cultured with the indicated concentrations of Y-27632 for 5 days. The experiment was performed using two HFK strains, with technical triplicates for each. Representative data shown are from HFK-2. HFK cell numbers per plate (HFKs) are shown on the left. A linear regression of the HFK cell numbers during log-phase growth was used to estimate the division rate (right). Division rates among HFKs cultured with the gradations in Y-27632 concentration were compared by a one-way ANOVA with Tukey's multiple comparisons test, which showed a significant increase in all conditions relative to vehicle (0 µM) and the standard conditional reprogramming concentration (10 µM Y-27632) (*P<0.05). (E) In a similar approach to D, RFP-labeled HFKs from three strains (HFK-2 shown here) were plated in technical triplicate with 10 µM Y-27632, then washed with PBS and cultured with the indicated Y-27632 concentrations for 48 h. RFP- and GFP-expressing cells were quantified with an Incucyte automated cell imager every 4 h (left). The S/G2/M fractions at 24 h were plotted (right) and compared to one another by a one-way ANOVA Tukey's multiple comparisons test, which showed a significant increase compared to vehicle (0 µM) or the standard conditional reprogramming concentration (10 µM Y-27632) (*P<0.05). Data in A,B,D and E are presented as mean±s.d.

Y-27632, at the 10 µM concentration used in conditional reprogramming, readily inhibited ROCK kinase activity. MLC2 (also known as MYL9), a known ROCK phosphorylation target (Riento and Ridley, 2003), served as an indicator of ROCK activity. Phosphorylated MLC2 (p-MLC2) levels increased 30 min to 1 h following Y-27632 removal from reprogrammed HFKs (Fig. 2A; Fig. S2C). After 1 h without Y-27632, re-addition of Y-27632 reversed MLC2 phosphorylation within minutes (Fig. 2B; Fig. S2D). The ROCKs could be activated in HFKs following Y-27632 washout, but under typical conditional reprogramming conditions, the ROCKs remained inhibited.

Gradations in Y-27632 concentration corresponded to gradations in MLC2 phosphorylation and HFK proliferation. We assessed a range of Y-27632 concentrations 3-fold and 10-fold lower or higher than the standard 10 µM used in conditional reprogramming (Chapman et al., 2014). Y-27632 decreased MLC2 phosphorylation in a concentration-dependent manner. The 10 µM Y-27632 concentration used in conditional reprogramming was the lowest concentration that reduced MLC2 phosphorylation by more than 50% (Fig. 2C; Fig. S2E). ROCK inhibition by Y-27632 led to a concentration-dependent increase in proliferation rates that paralleled the reduction in MLC2 phosphorylation, with Y-27632 concentrations of 10 µM and greater maximally increasing proliferation during log-phase growth within a single passage. (Fig. 2D) When we extended these studies to the Fucci system to assess cell cycle changes, the concentration-dependent increase in proliferation in the presence of Y-27632 also corresponded to a concentration-dependent increase in the S/G2/M proportion of HFKs (Fig. 2E).

### CRISPR-mediated deletion of ROCKs does not reprogram HFKs in the absence of Y-27632

The above results suggest that the contribution of Y-27632 to conditional reprogramming is through inhibition of one or both ROCKs. To test this mechanism of action, we genetically disrupted ROCK1 and ROCK2 via CRISPR-mediated knockout to determine whether loss of one or both ROCKs was able to reproduce the effects of Y-27632 treatment. Previous studies have reported that loss of ROCK2 prevents differentiation in immortalized cell lines that do not require Y-27632 to divide (Lock and Hotchin, 2009) and that ROCK2 inhibition promotes keratinocyte division (Roshan et al., 2016). We initially hypothesized that conditional reprogramming only requires inhibition of ROCK2 activity.

**Fig. 3.**
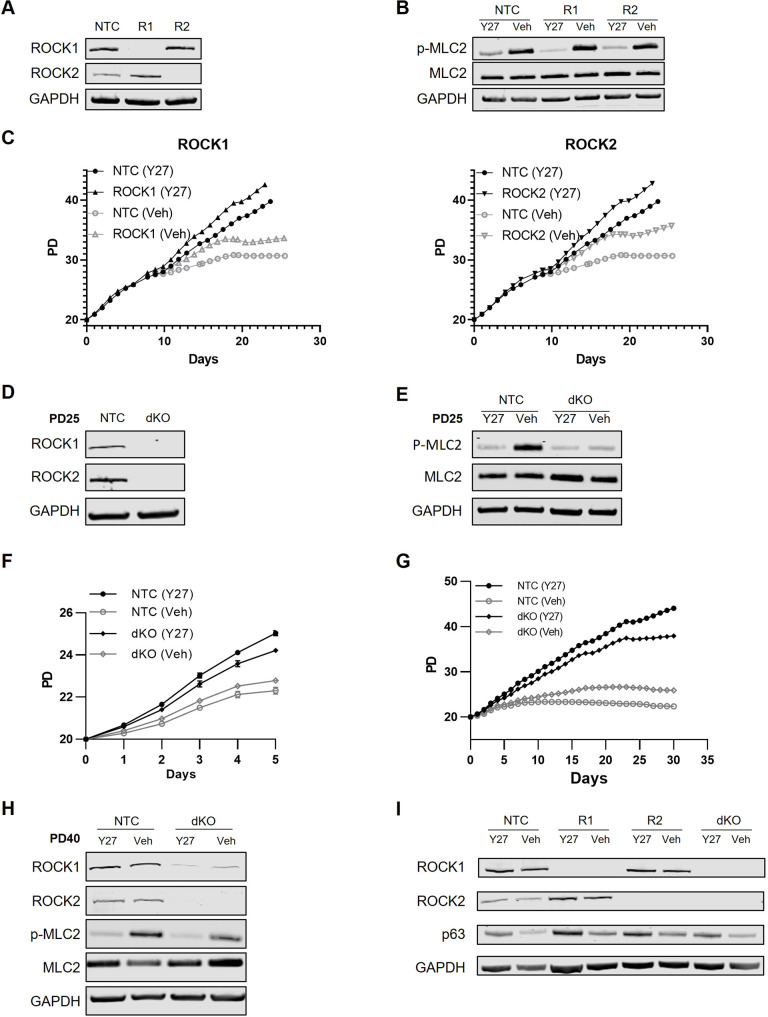
**CRISPR-mediated deletion of ROCKs does not reprogram HFKs without Y-27632.** (A) Protein was extracted from ROCK1-knockout (R1) and ROCK2-knockout (R2) HFKs, and from HFKs transduced with the NTC gRNA, within one passage of CRISPR-mediated knockout. Lysates were evaluated using western blotting, probing for ROCK1, ROCK2 or GAPDH as a loading control. The western blots shown, which used lysates from knockouts in the HFK-1 background, are representative of CRISPR-mediated knockouts performed in two biological replicates in each of three separate HFK strains. (B) Protein was harvested from ROCK1- or ROCK2-knockout HFKs, or from NTC HFKs, that had been cultured with Y-27632 before Y-27632 was maintained (Y27) or removed (Veh) for 1 h prior to lysis to allow ROCK activity. p-MLC2 and total MLC2 levels were evaluated by western blotting, where GAPDH served as an internal loading control. The western blots shown, which used lysates from knockouts in the HFK-1 background, are representative of the two biological replicates in each of three separate HFK strains. (C) Cultures of ROCK1-knockout (left) and ROCK2-knockout (right) HFKs, and of NTC HFKs, at 20 PDs were further cultured with (Y27) or without (Veh) Y-27632 for over 20 days, beginning on day eight of the experiment. Cell number was counted daily. The data shown, from knockouts in the HFK-1 background, are the mean of technical triplicates and are representative of five replicates across three HFK strain backgrounds. (D) Western blots probing for ROCK1 and ROCK2 were performed on lysates of control (NTC) and ROCK dKO HFKs shortly after CRISPR transduction (25 PDs) to confirm ROCK knockout, with GAPDH as a loading control. The western blots shown, which used lysates from strains in the HFK-2 background, are representative of two biological replicates in each of three HFK strains. (E) Protein lysates were harvested from NTC control or ROCK dKO HFKs shortly after CRISPR transduction (25 PDs), cultured either with (Y27) or without (Veh) Y-27632 for 1 h prior to lysis. To assess ROCK activity, western blots were probed for p-MLC2, total MLC2 and GAPDH as a loading control. The western blots shown, which used lysates from strains in the HFK-2 background, are representative of two biological replicates in each of three HFK strains. (F) Control (NTC) or ROCK dKO HFKs labeled with nucRFP were cultured for 5 days with (Y27) or without (Veh) Y-27632, beginning after 20 PDs. Total cell number was counted daily using an Incucyte automated cell imager to calculate the cumulative PDs of each culture, plotting the mean of three technical triplicates. Data are presented as mean±s.d. The data shown, which are from strains in the HFK-2 background, are representative of replicates across three HFK strains. (G) We extended the experiment described in F for 30 days with (Y27) or without Y-27632 (Veh), using control and ROCK dKO strains in the HFK-2 background. Total cell number was counted daily using an Incucyte automated cell imager to calculate the cumulative PDs of each culture, plotting the mean across three technical triplicates. Data are representative of this experiment performed across three HFK strains. (H) Protein lysates were harvested from NTC and ROCK dKO HFKs (HFK-2 background) that were cultured in conditional reprogramming conditions for 20 PDs following CRISPR-mediated knockout (total of 40 PDs), then cultured with (Y27) or without (Veh) Y-27632 for 1 h. To assess ROCK knockout and ROCK activity, western blots were probed with ROCK1, ROCK2, p-MLC2 and total MLC2 antibodies, with GAPDH as a loading control. The blots shown are representative of two replicates across three HFK strains. (I) Western blots were performed on protein lysates from control (NTC), ROCK1-knockout, ROCK2-knockout or ROCK dKO HFKs cultured in standard conditional reprogramming conditions (Y27) or 5 days after Y-27632 removal (Veh), within one passage following CRISPR–Cas9-mediated ROCK deletion (HFK-2 background). ROCK1 and ROCK2 antibodies served to validate ROCK isoform deletion. GAPDH served as an internal loading control, whereas ΔNp63 served as an epidermal stem cell marker. The blots shown are representative of two replicates across two HFK strains.

We generated CRISPR-mediated knockouts in three HFK strains to test whether single deletion of either ROCK isoform could substitute for Y-27632 in reprogramming of HFKs. Western blots showed that ROCK1 and ROCK2 were expressed in HFKs transduced with a non-targeting control (NTC) guide RNA (gRNA) but were absent from ROCK single-knockout cultures prepared in parallel (Fig. 3A; Fig. S3A).

We next tested whether ROCK isoform deletion altered ROCK activity in HFKs. Loss of either ROCK isoform alone did not reduce ROCK activity, suggesting that compensatory phosphorylation from the remaining ROCK isoform maintained MLC2 phosphorylation when either ROCK isoform was lost (Fig. 3B; Fig. S3B).

We tested whether single ROCK knockout could substitute for Y-27632 in conditional reprogramming. Despite the specific role reported for ROCK2 in keratinocyte differentiation, knockout of either ROCK2 or ROCK1 individually could not substitute for Y-27632. Single ROCK1 knockouts and single ROCK2 knockouts cultured without Y-27632 propagated slowly (Fig. 3C). When we examined conditional reprogramming marker expression in single ROCK isoform knockouts, only Y-27632-treated HFKs had consistently high levels of both CD44 and ΔNp63 (Fig. S3C). These data suggested that Y-27632 does not target a single ROCK isoform to drive conditional reprogramming.

As single-isoform ROCK knockouts required Y-27632 to proliferate, we next tested double deletion of the ROCK isoforms. Although double knockout (dKO) of the ROCK isoforms has been reported to be lethal in other systems (Kümper et al., 2016), we found that CRISPR-mediated ROCK dKO pools could be generated in conditionally reprogrammed HFKs cultured on collagen-1-coated plates. Western blots from lysates taken five PDs after transduction with CRISPR-containing lentivirus and selection showed that ROCK1 and ROCK2 were both expressed in HFKs transduced with the NTC gRNA but were absent from ROCK dKOs (Fig. 3D, Fig. S3D). Furthermore, dKO of the ROCKs greatly limited the increase in MLC2 phosphorylation following Y-27632 washout, which indicated that ROCK dKO prevented ROCK activity in keratinocytes, as expected (Fig. 3E; Fig. S3E).

We hypothesized that, as dKO of the ROCK isoforms prevented ROCK activity like Y-27632, the ROCK dKO keratinocytes would proliferate without Y-27632. When we evaluated proliferation over the first five PDs following CRISPR-mediated deletion, ROCK dKO HFKs proliferated almost as slowly as control HFKs when Y-27632 was removed, whereas addition of Y-27632 allowed them to propagate rapidly (Fig. 3F; Fig. S3F). This surprising result suggested that the short-term effect of Y-27632 on proliferation depends at least in part on inhibiting targets other than ROCKs.

ROCK dKO HFKs without Y-27632 ceased proliferating after 15 days (Fig. 3G; Fig. S3G). Continued passage of ROCK dKO HFK pools led to the restoration of Y-27632-sensitive MLC2 phosphorylation, which likely included a contribution from the increased ROCK1 protein expression (Fig. 3H; Fig. S3H). Thus, we could not rule out some longer-term effects of Y-27632 acting directly via ROCK inhibition. However, we examined epidermal stem cell marker expression in HFKs within the first passage while the ROCK dKO HFK cultures still maintained low ROCK protein levels. We observed that HFKs lacking either or both ROCK isoforms had increased levels of ΔNp63 in cultures with Y-27632 (Fig. 3I). This suggested that activity from Y-27632 beyond inhibiting ROCKs was required for epidermal stem cell marker expression in conditionally reprogrammed HFKs.

### Other ROCK inhibitors differ from Y-27632 in their ability to promote conditional reprogramming

As another approach to evaluate whether Y-27632 has effects other than inhibition of ROCKs that promote conditional reprogramming, we exploited the diversity in chemical structures, *in vitro* binding affinities and off-target profiles among other compounds developed as ROCK inhibitors (Feng et al., 2016). We evaluated ROCK inhibitors previously reported to reprogram HFKs (Chapman et al., 2014) or promote HFK proliferation in short-term assays (Zhang et al., 2018).

**Fig. 4.**
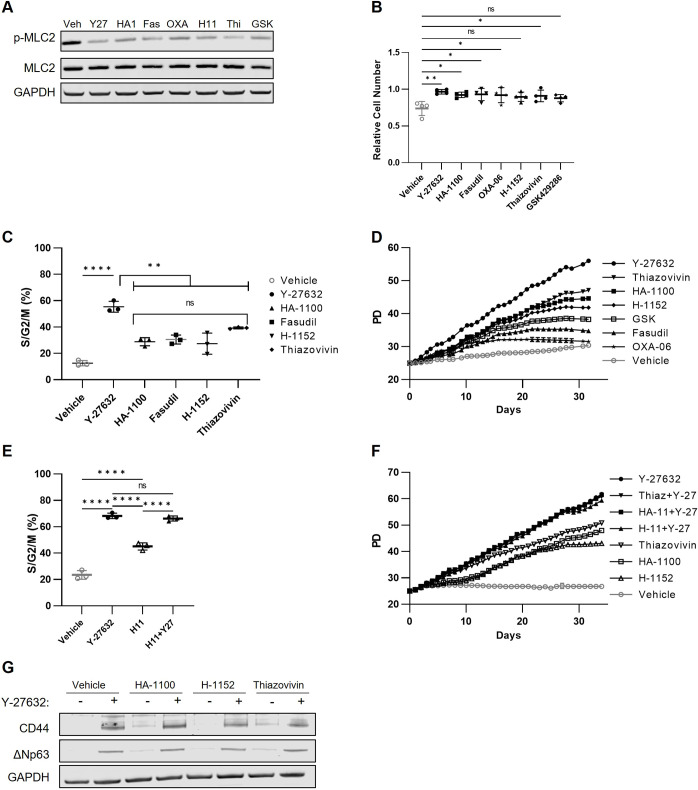
**Other ROCK inhibitors differ from Y-27632 in their ability to promote conditional reprogramming.** (A) Protein lysates were harvested from HFKs (HFK-6) that were either treated with the indicated ROCK inhibitor for 1 h (Y27, 10 µM Y-27632; HA1, 20 µM HA-1100; Fas, 20 µM fasudil; OXA, 3 µM OXA-06; H11, 1 µM H-1152; Thi, 10 µM Thiazovivin; GSK, 100 nM GSK429286) or cultured in the absence of Y-27632 for 1 h (Veh) to allow ROCK activity. Blots were probed for p-MLC and total MLC2, with GAPDH used as a loading control. Blots shown are representative of two replicates across three independent HFK strains. (B) HFKs were plated following trypsinization with vehicle, Y-27632, or the other ROCK inhibitors as listed in A. The viable HFK number was counted across four independent strains every 3 h over the first over 36 h after plating, with six technical replicates per strain. The relative adherent HFK number at 24 h, relative to the initial cell number, is plotted in this graph, where each point represents the mean relative cell number for each of the four HFK strains. We compared the mean±s.d. relative adherent HFK numbers across all four strains for the vehicle control with each of the other treatments using a one-way ANOVA with Tukey's multiple comparisons test (*P<0.05; **P<0.01; ns, not significant). (C) Fucci-labeled HFKs were plated with Y-27632 to allow equal cell adhesion. At 24 h after plating the HFKs, the medium containing Y-27632 was removed, HFKs were washed with PBS, then medium containing vehicle, Y-27632, HA-1100, fasudil, H-1152 or Thiazovivin, as in A, was added. The cell cycle distribution was tracked via automated fluorescence imaging using an Incucyte imager by counting the RFP- and GFP-labeled HFKs every 4 h in technical triplicates. The S/G2/M fraction in one of four representative HFK strains (HFK-6) at 24 h is plotted. Statistical significance was evaluated using a one-way ANOVA with Tukey's multiple comparisons test (**P<0.01; ****P<0.0001; ns, not significant). (D) Four HFK strains, starting after 25 PDs, were cultured for 35 days with each ROCK inhibitor (as listed in A), in technical triplicates, with data from one strain (HFK-6) plotted here. (E) Fucci-labeled HFKs were plated with Y-27632 to allow equal cell adhesion, with subsequent washout as described above. HFKs were cultured in medium containing 10 µM Y-27632, vehicle, 1 µM H-1152, or a combination of both 10 µM Y-27632 and 1 µM H-1152 (H11+Y27), in technical triplicate in three HFK strains (data from HFK-6 shown here). The cell cycle fraction was tracked by counting RFP- and GFP-labeled HFKs every 4 h with the Incucyte imager for 36 h. The S/G2/M fraction at 24 h was specifically evaluated to test for differences using a one-way ANOVA with Tukey's multiple comparisons tests (****P<0.0001; ns, not significant). (F) HFK cultures at 25 PDs were further cultured for 30 days with the indicated ROCK inhibitors (Thiaz, 10 µM Thiazovivin; HA11, 20 µM HA-1100; H-11, 1 µM H-1152) either with or without 10 µM Y-27632. Cultures were scanned daily in the Incucyte imager to count the RFP-labeled cells in technical triplicates per strain. PDs were calculated from the daily cell counts and plotted over time. Here, data from one of four strains (HFK-6) are plotted. (G) HFKs were cultured with the listed ROCK inhibitors (HA-1100, H-1152 or Thiazovivin), as in F, with or without 10 µM Y-27632 for 5 days. Protein was then extracted and used for western blots probed for CD44 and ΔNp63, with GAPDH as a loading control. The blots shown, which used samples from HFK-6, are representative of the results from four strains. Data in B–F are plotted as mean±s.d.

First, we tested whether the ROCK inhibitors disrupted ROCK activity in HFKs, as measured by MLC2 phosphorylation. All the ROCK inhibitors we tested – HA-1100, fasudil, OXA-06, Thiazovivin, H-1152 and GSK429286 – prevented MLC2 phosphorylation at concentrations previously reported to result in reprogramming (Chapman et al., 2014; Zhang et al., 2018) (Fig. 4A; Fig. S4A). As with Y-27632, increasing gradations beyond these concentrations did not further reduce levels of p-MLC2 (Fig. S4B,D). These ROCK inhibitors rapidly decreased MLC2 phosphorylation in timecourses similarly to Y-27632 (Fig. S4C).

Having documented that these ROCK inhibitors decreased MLC2 phosphorylation, we evaluated whether ROCK inhibition contributes to different aspects of conditional reprogramming. First, we observed that addition of the ROCK inhibitors resulted in a greater number of adherent HFKs relative to that in control cultures following plating, similarly to addition of Y-27632 (Fig. 4B). This result supports a direct role for ROCKs in the adherence aspect of conditional reprogramming.

We next used the Fucci system to test whether these ROCK inhibitors increased the fraction of HFKs in S/G2/M similarly to Y-27632. No other ROCK inhibitor increased the S/G2/M fraction as extensively as Y-27632 did, each providing only a modest increase in the S/G2/M fraction relative to the control (Fig. 4C). As with MLC2 phosphorylation, increases in ROCK inhibitor concentrations beyond those reported to conditionally reprogram did not further increase the fraction of HFKs in S/G2/M (Fig. S4F–H).

We then evaluated whether the ROCK inhibitors could promote long-term propagation of HFKs, as observed during conditional reprogramming with Y-27632, using concentrations that maximally decreased MLC2 phosphorylation. All of the ROCK inhibitor treatments provided a proliferative advantage over HFKs cultured with vehicle, yet none of the ROCK inhibitors enabled HFKs to propagate as successfully as did Y-27632 (Fig. 4D; Fig. S4E).

To test the hypothesis that other ROCK inhibitors lacked a function unique to Y-27632, we tested whether Y-27632 could rescue proliferation when ROCKs were already inhibited by another ROCK inhibitor. When Y-27632 was added to HFKs treated with other ROCK inhibitors as in Fig. 4C, HFKs reached the higher S/G2/M fraction seen with Y-27632 alone (Fig. 4E; Fig. S4I). We found that Y-27632 could promote long-term proliferation when added to HFKs cultured with other ROCK inhibitors (Fig. 4F). Furthermore, Y-27632 treatment increased the expression of the stem cell markers CD44 and ΔNp63 in HFK cultures, whereas treatment of HFKs with the other ROCK inhibitors did not increase the levels of these markers to those seen following Y-27632 addition (Fig. 4G). These data indicate that, although ROCK inhibition plays a part in conditional reprogramming, some effect of Y-27632 beyond ROCK inhibition promotes proliferation and stemness in keratinocyte cultures.

## DISCUSSION

Using a primary keratinocyte model, we identified several effects of Y-27632 that promote the replicative immortality required by conditional reprogramming. Y-27632 maintained viable HFKs when the cells were initially plated on culture dishes, as did other ROCK inhibitors previously reported to support conditional reprogramming. However, maintaining HFK viability and adherence after plating was insufficient for conditional reprogramming. Adherent HFKs still required Y-27632 to promote cell cycle progression. Nevertheless, simple stimulation of proliferation does not explain the irreversible cell cycle exit after 4 days without Y-27632. ΔNp63 and CD44 levels decreased when Y-27632 was removed from conditionally reprogrammed HFKs. Our findings are consistent with other groups that have shown that Y-27632 blocks differentiation (Chapman et al., 2014). It remains unknown whether Y-27632 directly inhibits a differentiation program or prevents differentiation by maintaining HFKs in a stem-cell-like state poised for continued proliferation in conditional reprogramming.

Conditional reprogramming via Y-27632 decreased ROCK activity in HFK cultures. We demonstrated the direct correlation between Y-27632-dependent HFK proliferation, cell cycle progression and decreased MLC2 phosphorylation. However, these correlative data were insufficient to determine whether ROCK inhibition directly plays a role to conditionally reprogram HFKs.

We directly tested the role of ROCK-specific inhibition in conditional reprogramming using CRISPR-mediated deletion of ROCKs from HFKs. As the presence of a single ROCK isoform could maintain ROCK activity in HFKs, it was unsurprising that ROCK single-knockout HFKs required Y-27632 to be conditionally reprogrammed. Nevertheless, following double deletion of the ROCK isoforms, which prevented ROCK activity, HFKs still required Y-27632 for rapid proliferation. These results suggest that Y-27632 targets something other than or in addition to ROCKs to promote conditional reprogramming. We could not exclude the possibility that ROCK activity must be inhibited along with additional targets for conditional reprogramming. Slight ROCK1 expression was present when ROCK dKO cells were propagated more than 20 PDs after CRISPR deletion, presumably due to the selective outgrowth of rare cells with incompletely knocked-out ROCK1. This indicates a proliferative disadvantage of the ROCK dKO cells, consistent with other reports of ROCK dKO lethality (Kümper et al., 2016). We thus could not use the ROCK dKO pools as a model in longer-term studies to identify Y-27632 targets besides ROCKs. This apparent selection against ROCK dKO, which is insufficient to promote conditional reprogramming, can be considered further indirect evidence for an additional, ROCK-independent effect of Y-27632 that promotes conditional reprogramming. Early passage ROCK dKO HFK cultures, in which we documented that both ROCK1 and ROCK2 were deleted, had decreased levels of the epidermal stem cell marker ΔNp63 when Y-27632 was removed. This indicates that the key epidermal stem cell renewal supported by Y-27632 requires activity besides ROCK inhibition alone.

The ability of ROCK inhibitors other than Y-27632 to reprogram HFKs has been used as evidence that specific, on-target ROCK inhibition promotes conditional reprogramming. We confirmed that other ROCK-targeting compounds inhibited ROCK activity similarly to Y-27632. However, ROCK inhibitors other than Y-27632 did not support HFK propagation nearly as well as Y-27632, even at optimal concentrations. No other ROCK inhibitor increased the fraction of HFKs in S/G2/M to the extent of Y-27632. Y-27632, when added to the other ROCK inhibitors, could further increase the fraction of HFKs in S/G2/M. Finally, whereas all ROCK inhibitors decreased ROCK activity, no other ROCK inhibitor increased CD44 and ΔNp63 levels in HFK cultures to the levels observed following addition of Y-27632.

As the other ROCK inhibitors enhanced HFK adhesion and provided some proliferation advantage, ROCK inhibition presumably plays a role in conditional reprogramming. Nevertheless, as none of the other ROCK inhibitors could completely substitute for Y-27632, our results suggest that targets of Y-27632 other than ROCKs must also be inhibited for the most effective conditional reprogramming. For example, our proliferation results are consistent with the hypothesis that direct ROCK inhibition promotes proliferation, yet some effect of Y-27632 on other targets was necessary to prevent HFK differentiation and promote expression of stem cell markers. Future research efforts should characterize how Y-27632 promotes stem cell marker expression, if not through ROCKs.

The primary HFK model was advantageous for our study because it precluded any confounding mutations that would be present in cancer cell models and was the same model system used in most studies on conditional reprogramming. We do not expect that the limitation of this study to male epithelial cells will affect the interpretation of our results, as Y-27632 can readily conditionally reprogram cultures from male or female epithelial cells (Chapman et al., 2010). Future studies can investigate potential sex-specific differences.

In this study, we focused on the effects of Y-27632 on bulk HFK cultures, rather than on a single-cell basis. A single-cell approach should be used in future studies to investigate whether Y-27632 promotes a stem cell state in a subset or all HFKs, as other studies have explored (Roshan et al., 2016). Beyond evaluating the role of Y-27632 in promoting epidermal stem cell marker expression, further studies should also investigate the tissue relevance of stem cell marker expression in cultured cells compared to stem cells in the epidermis.

Further studies should also be performed in other systems that use Y-27632 in order to test to what extent ROCK inhibition specifically accounts for the functions of Y-27632. Y-27632 has been used in induced pluripotent stem cell culture to promote viability during cell cryopreservation (Watanabe et al., 2007). This effect of Y-27632 appears to be dependent upon ROCK inhibition (Chen et al., 2010). Other uses for Y-27632 include its role in chemical-induced cellular reprogramming cocktails that generate totipotent (Yang et al., 2022) or pluripotent (Breunig et al., 2021) stem cells, and facilitation of reprogramming into other cell types (Kim et al., 2020). Although the role of Y-27632 in controlling cell fate is currently attributed to its activity on ROCK, (Kim et al., 2020) our results suggest that the ability of Y-27632 to promote epidermal stem cells involves a target other than ROCKs. Understanding the ROCK-independent action of Y-27632 could yield insights into the core regulatory processes that control cell fate.

## MATERIALS AND METHODS

### Foreskin processing

Primary keratinocyte and fibroblast cultures were established from eight neonatal foreskins provided by the National Cancer Institute (NCI) Cooperative Human Tissue Network (CHTN). The epidermal and dermal layers of each foreskin were separated from one another via overnight dispase digestion followed by mechanical separation, as previously described (Aasen and Belmonte, 2010). For each foreskin, a keratinocyte strain and a fibroblast strain were established from the epidermal and dermal layers, respectively. Keratinocytes were isolated by gentle trypsinization (0.05% trypsin-EDTA at 37°C for 1 h) of the epidermal layer and plating the resultant single-cell suspensions on tissue culture dishes. The eight keratinocyte strains, each derived from an independent donor, were designated HFK-1 to HFK-8. Fibroblast cultures grew out from tissue fragment explants plated directly on tissue culture dishes after 1–2 weeks. The keratinocyte or fibroblast strains from individual foreskins were kept separate from other keratinocytes or fibroblasts. Keratinocyte strains and fibroblast strains were cultured as described below.

HFK strains were maintained in conditional reprogramming conditions, discussed below, immediately after initial dissociation from tissue. Short-tandem repeat (STR) analysis, described below, confirmed that the strains established in the study were distinct from one another and from established cell lines. HFK cultures showed the expected morphology and expressed epithelial keratins and collagen markers (Fig. S1). The human foreskin fibroblast (HFB) strains we also isolated from these foreskin donors served as controls in the epithelial marker studies.

Primary keratinocyte and fibroblast cultures were established from eight neonatal foreskins, otherwise to be discarded, provided by the National Cancer Institute (NCI) Cooperative Human Tissue Network (CHTN) without identifying information, under conditions deemed by the Ohio State University Institutional Review Board not to constitute human subjects research.

### Cell culture

All mammalian cells were cultured in humidified tissue culture incubators (37°C, 5% CO_2_; Heracell VIOS 160i). All cell culture work was performed in Thermo Scientific 1300 Series A2 biosafety cabinets. The incubators were set to different oxygen concentrations, depending upon the cell type in culture. Most cells in this study, including HFKs, were cultured in 3% oxygen to mimic physiological conditions, created by additional gaseous nitrogen in the incubators. Other cell types were cultured with atmospheric oxygen. All cultured cells were pelleted during passaging via centrifugation at 200 ***g*** in a table-top centrifuge. Cell cryopreservation was performed in the cell-type appropriate culture medium (listed below) containing 10% DMSO, and cells were gradually cooled to −80°C overnight in isopropanol-containing plastic containers, then stored in liquid nitrogen (<−130°C) for long-term storage. All established cell lines or newly generated cell strains used in this study were genotyped via STR profiling to confirm individual identity by the Genomics Core of The Ohio State University Comprehensive Cancer Center (OSUCCC). All genomic DNA for STR profiling was isolated with the DNeasy Blood & Tissue Kit (Qiagen). All mammalian cells in culture routinely tested negative for the presence of mycoplasma (Lonza). All ROCK inhibitors used in this study were reconstituted as described in Table S1. Details of cell culture reagents are given in Table S5.

#### 293FT cells

293FT cells (Life Technologies), cultured in atmospheric oxygen, were used for lentiviral production. Routine culture was in high-glucose DMEM containing 10% FBS, 1% penicillin-streptomycin-glutamine. Lentiviral production used the same DMEM formulation containing an additional 1 mM sodium pyruvate (Gibco).

#### Human foreskin fibroblasts

The HFB strains described above were cultured in high-glucose DMEM (Gibco) containing 10% FBS and 1% penicillin-streptomycin. HFBs were grown in incubators at atmospheric oxygen. HFBs were subcultured with 0.05% trypsin-EDTA, with trypsin inactivated after 3 min with a volume of culture medium equal to the volume of trypsin used.

#### Human foreskin keratinocytes

Conditionally reprogrammed HFKs were routinely cultured in conditioned medium, as described below, in 3% oxygen with 10 µM Y-27632 (Enzo), unless otherwise stated. HFKs were subcultured via digestion with 0.25% trypsin-EDTA, which was inactivated with a volume of soybean trypsin inhibitor solution equal to the volume of trypsin solution added. The soybean trypsin inhibitor solution was 250 µg/ml soybean trypsin inhibitor (Sigma-Aldrich) in PBS (Gibco), made sterile by filtration through a 0.22 µm PVDF filter.

We used the population doubling (PD) method to track the relative age of each HFK culture, calculated as PD=log_2_(final cell number)−log_2_(initial cell number), cumulatively added each passage. HFKs were routinely re-plated at a 1:32 split, ∼2.055×10^3^ HFK/cm^2^, such that five PDs would result in 80% confluent dishes.

#### Conditioned medium and feeder fibroblasts for HFK conditional reprogramming cultures

HFK cultures require a medium conditioned by irradiated feeder fibroblasts to be conditionally reprogrammed (Liu et al., 2017). To make conditioned medium, first fibroblasts were expanded then irradiated. Second, irradiated fibroblasts were plated in HFK medium to be conditioned, which was harvested every 48 h for two total collections.

##### Feeder 3T3-J2 fibroblast culture and irradiation

The murine fibroblast strain 3T3-J2 was a gift from James Rheinwald (Dermatology Department at Brigham and Women's Hospital, Boston, MA, USA). 3T3-J2 fibroblasts were cultured in DMEM containing 10% bovine calf serum (BCS) and 1% penicillin-streptomycin, at atmospheric oxygen. 3T2-J2 cells were subcultured via 0.05% trypsin-EDTA, which was inactivated with a volume of culture medium equal to the volume of trypsin-ETDA solution used.

3T3-J2 fibroblasts were irradiated with an X-ray irradiator (Radsource 2000). 3T3-J2 cells were trypsinized into a single-cell suspension and irradiated at 30 Gy. Irradiated 3T3-J2 (3T3-J2+IR) cells were functionally validated to be senescent by demonstrating that the 3T3-J2+IR cells failed to divide, compared to non-irradiated 3T3-J2s, over 5 days in standard 3T3-J2 culture conditions. Immediately after irradiation, 3T3-J2+IR cells were cryopreserved.

##### Conditioned medium

HFKs were cultured in the following conditioned medium. HFK 3F:1D medium, the base medium conditioned by 3T3-J2+IR feeder fibroblasts, contained three parts Ham's F-12 to one part DMEM by volume and the supplements listed in Table S2. 3T3-J2+IR fibroblasts were thawed from liquid nitrogen storage, pelleted to remove freezing medium, then plated in HFK 3F:1D medium at 4×10^4^ 3T3-J2+IR cells per cm^2^, typically in five-layer flasks (3.5×10^7^ cells on 875 cm^2^ with 150 ml medium). HFK 3F:1D medium was conditioned by 3T3-J2+IR fibroblasts at atmospheric oxygen. After 48 h, a first conditioned medium batch was collected and then stored at 4°C, and fresh non-conditioned HFK 3F:1D was added to the dishes. After an additional 48 h, a second conditioned medium batch was harvested. The two batches were pooled together and diluted 3:1 with non-conditioned HFK 3F:1D medium, or three parts conditioned medium per one part non-conditioned medium by volume. The conditioned medium was sterile filtered, first through a 0.45 µm PVDF filter then through a 0.22 µm PVDF filter, aliquoted and frozen at −80°C for long-term storage. To prevent any decline in function, frozen conditioned medium was always used within 6 months, and thawed conditioned medium was never refrozen and was used within 2 weeks.

### Western blotting

Typical cell lysis was with an SDS-based buffer (4% SDS in H_2_O). Protein was extracted by scraping adherent cells with a disposable cell lifter, after adding a volume of lysis buffer appropriate for the dish surface area, and transferring to an Eppendorf tube. Protein lysates were sonicated with 30 pulses (Misonix XL-2000; setting 3). Protein concentration was estimated via a colorimetric Bradford assay (DC Protein Assay, Bio-Rad), which was quantified with an Epoch2 microplate reader (BioTek; 750 nm). 4× protein loading buffer (LI-COR) containing 5% dithiothreitol (Sigma-Aldrich) was added to protein lysates such that the final concentration of loading buffer was ≥1×. Across a given experiment, the final protein concentration was equalized by adjusting the final volume of loading buffer added. Lysates were heated at 95°C for 5 min.

Protein lysates were loaded into precast 4–12% Bis-Tris gels (Invitrogen), with 20 µg of total protein per lane. PAGE was conducted at a constant voltage (200 V, 0.5 A, 25 min; Labnet Enduro 300 V power supply) in MES (Invitrogen). Protein was transferred to membranes using a semi-dry approach with a discontinuous buffer system. The discontinuous buffer system included Anode Buffer #1 (0.3 M Tris, 10% methanol), Anode Buffer #2 (25 mM Tris, 10% methanol), and Cathode Buffer (25 mM Tris, 192 mM glycine, 10% methanol) all at pH 10.4. To allow use of a fluorescent detection system, we transferred to 0.2 µm PVDF membranes (Thermo Fisher Scientific). Each western blot transfer was layered, from bottom (anode) to top (cathode), with extra thick blot paper (Bio-Rad) equilibrated in Anode Buffer #2, a PVDF membrane activated with 100% methanol (Sigma) and equilibrated in Anode Buffer #1, the Bis-Tris gel equilibrated in Cathode Buffer, and finally an additional sheet of extra thick blotting paper equilibrated in Cathode Buffer. Semi-dry transfer was conducted at a constant voltage (25 V, 1.0 A, 30 min; Trans-Blot Turbo Transfer System, Bio-Rad). Following transfer, membranes were briefly rinsed with 1× TBS (Bio-Rad) and dried between two pieces of new blot paper for at least 10 min, until dry. Then membranes were reactivated by a brief incubation with 100% methanol, rinsed with TBS for 5 min, then incubated in Intercept TBS Blocking Buffer (LI-COR) for 1 h. Membranes were incubated with primary antibodies overnight at 4°C in Intercept TBS Blocking Buffer containing 0.2% Tween-20, at the listed antibody dilutions in Table S3. After overnight incubation, membranes were washed three times in 5 min washes with TBST (1×TBS containing 0.05% Tween-20). Membranes were incubated with a species-appropriate fluorescent secondary antibody (LI-COR) in Intercept TBS Blocking Buffer containing 0.2% Tween-20 and 0.02% SDS for 1 h at room temperature, washed three times with TBST, dried between blot filter paper, then imaged in an Odyssey CLx Imager (LI-COR). Further analyses or quantification were performed with the Image Studio software (LI-COR). Please see Figs S5 and S6) for uncropped versions of western blots. Details of western blotting reagents and antibodies can be found in Table S5.

### Lentiviral preparation and concentration

Lentiviruses were produced via transfection into the 293FT packaging cell line. Plasmid DNA was transfected into 293FT with polyethylenimine (PEI). Lentiviral transfer vectors were co-transfected with psPAX2 (Addgene plasmid 12260, deposited by Didier Trono) as the packaging plasmid and pVSV-G (Addgene plasmid 8454, deposited by Bob Weinberg) as the envelope plasmid. Plasmids were added at a ratio of 10 transfer vector:10 psPAX2:1 pVSV-G by mass, scaled by culture dish area. Plasmid DNA was diluted in OPTI-MEM serum-free medium (Gibco). Three volumes 1 mg/ml PEI was added per µg of total plasmid DNA, incubated at room temperature for 30 min, then added dropwise onto 293FT cells. The virus-containing medium was harvested at 48 h and 72 h after transfection. Both collections were pooled together and concentrated with a PEG-NaCl solution (30% PEG-8000 solution in 1.6 M NaCl).

Virus-containing medium from transfected 293FT cells was filtered through a 0.45 µm PVDF filter to remove cells and debris. 0.4 volumes of PEG-NaCl were added and chilled at 4°C overnight. Viral particles were pelleted by centrifugation at ∼2500 ***g*** for 30 min at 4°C. The supernatant was discarded, and the viral pellet was resuspended in a volume of PBS that was one-tenth the volume of viral harvest medium, to concentrate 10-fold. 48 h and 72 h harvests were pooled together. Viruses were aliquoted to prevent multiple freeze–thaw cycles and were stored at −80°C. Details of reagents can be found in Table S5.

### Lentiviral transduction

All HFKs infected with lentiviruses were transduced by spin infection with polybrene (hexadimethrine bromide; Sigma-Aldrich). Concentrated virus was added along with 5 µg/ml polybrene, then plates were spun at ∼1250 ***g*** for 1 h at room temperature. Medium was changed the first day after infection. Infected cells were split on the second day after infection to new dishes. Selection was applied, when appropriate, on the third day following infection, to allow time for lentiviral integration and expression. Details of reagents can be found in Table S5.

### Mammalian selectable markers

Appropriate concentrations for selectable markers were experimentally determined on an individual cell strain basis. Mammalian selectable markers included blasticidin (Gibco), hygromycin B (Gibco), puromycin (Sigma), Geneticin G418 (Thermo Fisher Scientific) and Zeocin (Invivogen). In each case, selection was applied until complete cell death in a parallel negative control for transduction or transfection that confirmed selection efficacy.

### RNA isolation with TRIzol

RNA was isolated with TRIzol reagent (Invitrogen), as described by the manufacturer. Per 10 cm^2^ dish, 1 ml of TRIzol was used to lyse cells for 5 min. Cellular material was phase separated by transferring the TRIzol lysate to an Eppendorf tube, mixing with 200 µl chloroform (Sigma-Aldrich), resting for 3 min, then centrifuging at 4°C for 15 min at ∼10,000 ***g***. The clear, top-most phase containing RNA was carefully pipetted (∼400 µl) to a new microfuge tube. The RNA was precipitated by adding 340 µl isopropanol (Sigma-Aldrich), letting the mixture sit for 10 min and then centrifuging at 4°C for 10 min at ∼10,000 ***g***. The RNA pellet was then washed by gently aspirating off the fluid, mixing with 800 µl 75% ethanol (Sigma-Aldrich) and centrifuging at 4°C for 5 min at ∼7600 ***g***. The RNA pellet was air dried following the removal of ethanol. The RNA was solubilized by reconstituting the pellet in 20 µl nuclease free water (Invitrogen) and incubating at 55°C for 10 min. RNA was stored at −80°C. Details of reagents can be found in Table S5.

### cDNA synthesis

After isolation, total RNA was quantified with a spectrophotometer (BioTek Take3 Trio), residual DNA was digested, and RNA was reverse transcribed into cDNA. RNA samples were DNase treated with the RNA Clean & Concentrator-5 containing DNase (Zymo Research), per the manufacturer's instructions. cDNA was synthesized with the High-Capacity cDNA Reverse Transcription Kit (Applied Biosystems) per the manufacturer's instructions, with 2 µg of total RNA input per 20 µl reaction such that the final concentration after reverse transcription was 100 ng cDNA/µl. Thermal cycler (Bio-Rad) conditions were 10 min at 25°C, 120 min at 37°C, 5 min at 85°C, followed by a brief hold at 4°C. cDNA was stored at −80°C. Details of reagents can be found in Table S5.

### qPCR

qPCR of reverse-transcribed cDNA was performed with the SsoAdvanced Universal SYBR Green Supermix (Bio-Rad), as described by the manufacturer, in a Bio-Rad CFX384 instrument. A typical 10 µl reaction comprised 5 µl SsoAdvanced Universal SYBR Green Supermix (2×), 1 µl cDNA (diluted to 1 ng cDNA/µl), 0.5 µl each forward and reverse primer (500 nM) and 3 µl nuclease-free H_2_O. Specific primer sequences are listed in Table S4. Details of reagents can be found in Table S5. All qPCR experiments were performed in technical triplicate. The thermal cycler was set to the PrimePCRMetl384 protocol (Bio-Rad), or 2 min at 95°C then 40 cycles of 5 s at 95°C followed by 30 s at 60°C. The melt curve cycled from 5 s at 95°C then ramped up from 65°C to 95°C at a rate of 0.5°C/5 s. Further analysis was performed with the CFX Maestro Software (Bio-Rad).

### Molecular cloning and bacterial work

All plasmid expansion was performed in Stbl3 bacteria (Invitrogen). Liquid bacterial cultures were grown in a shaking bacterial incubator (New Brunswick I24) at 37°C and 225 r.p.m. Liquid bacterial cultures were grown with Luria–Bertani (LB) broth, containing the appropriate selection marker when necessary. Bacterial agar plates were cultured in a standard bacterial incubator (Labnet) at 37°C. Bacteria were cultured on LB-agar plates made by autoclaving LB with agar and pouring into bacterial dishes to solidify, with appropriate antibiotics added once the mixture cooled to 60°C. Electrocompetent Stbl3 bacteria were generated as described previously (Sambrook et al., 1989).

Plasmid DNA was electroporated (Bio-Rad MicroPulser) into 50 µl of electrocompetent Stbl3 bacteria (1.0×10^10^ cells, 0.2 cm cuvettes, Ec2 pulse setting) then recovered in 1 ml SOC medium (Howland, 1995) for 1 h at 37°C before plating on LB-agar plates containing the appropriate selection marker. Plasmid DNA for further subcloning was extracted from 3 ml miniprep cultures with QIAprep Spin Miniprep Kit (Qiagen 27106). Plasmid DNA for transfection into mammalian cells was extracted from 100 ml midiprep cultures with the NucleoBond Xtra Midi Plus EF kit (Macherey-Nagel). Details of reagents can be found in Table S5.

### LentiCRISPRv2 subcloning

ROCK1- or ROCK2-targeting gRNAs or a control gRNA were cloned into versions of LentiCRISPRv2 subcloned to confer either blasticidin or hygromycin resistance. LentiCRISPR v2 was from Addgene (deposited by Feng Zhang; Addgene plasmid 52961; RRID: Addgene_52961) and was further subcloned to express blasticidin or hygromycin resistance. We abolished the MluI restriction enzyme cut site immediately upstream of the CMV enhancer sequencing using DNA mutagenesis PCR to change a guanine (G14488 in the Addgene sequence 244694) to a thymidine. The MluI cut site downstream of the puromycin resistance cassette became a unique cut site that we used to clone in other resistance gene cassettes. LentiCRISPRv2 was digested with a MluI and BamHI digestion to drop out the puromycin cassette. Blasticidin- and hygromycin-resistance genes were cut out of LentiLox-Blast (LLB) and LentiLox-Hygro (LLH) [subcloned from pLL3.7 (Addgene plasmid 11795, deposited by Luk Parijs), in which the eGFP coding sequence was replaced with blasticidin- and hygromycin-resistance genes] via MluI and BamHI digestion. The LentiCRISPRv2 fragment or resistance gene cassette containing fragments were gel purified following agarose gel electrophoresis with the QIAEX II Gel Extraction Kit (Qiagen). LentiCRISPRv2 and resistance cassette fragments were ligated with ElectroLigase (NEB) and electroporated into bacteria, and positive clones were identified via Sanger sequencing. ROCK1- and ROCK2-targeting gRNAs (ROCK1, 5′-GAAGTATTAAAATCCCAAGG-3′; ROCK2, 5′-TAGTAGGTAAATCCGATGAA-3′) were designed with CRISPick (formerly GPP sgRNA Designer; Doench et al., 2016; Sanson et al., 2018; https://portals.broadinstitute.org/gppx/crispick/public) then annealed and cloned into the modified versions of LentiCRISPRv2 with blasticidin or hygromycin resistance cassettes, as described by the Zhang lab (Sanjana et al., 2014). The control gRNA used (NTC: 5′-ACGGAGGCTAAGCGTCGCAA-3′) was a non-targeting control gRNA from a published genome-wide CRISPR screen (Sanson et al., 2018). In brief, the gRNA sequence-containing primers were annealed then ligated with ElectroLigase into a gel purified fragment of LentiCRISPRv2 after BsmBI digestion to drop out the 2 kb spacer fragment. Positive clones were validated by Sanger sequencing across the gRNA site. Details of reagents can be found in Table S5.

### Incucyte-compatible fluorescent strain labeling and analysis

HFKs were labeled with a stably expressed, nuclear-localized red or green fluorescent protein (nucRFP or nucGFP, respectively) to aid in automated cell counting in an Incucyte S3 with a green–red optical configuration [green excitation (ex), 460 nm with 440–480 nm passband; green emission (em), 524 nm with 504–544 nm passband; red ex, 585 nm with 565–605 nm passband; red em, 635 nm with 625–705 nm passband]. The RFP used in the below systems was mKate2 (ex, 588 nm; em, 633 nm), whereas the GFP was tagGFP2 (ex, 483 nm; em, 506 nm). All images were analyzed in the Incucyte S3 software (Sartorius), as described below.

HFKs labeled with a nucRFP for cell counting were infected with lentiviruses purchased from the manufacturer (Sartorius) and transduced using our lentiviral transduction protocol described above. Stable cell lines were generated via selection, as described above, where fluorescent protein expression was driven by an EF1α promoter. Compatible culture plates were scanned in the Incucyte S3 with the ‘Whole Well’ scan type, to ensure that all cells in each well were counted. Standard phase-contrast images and red fluorescence images (400 ms acquisition) were collected with the 4× objective. Red fluorescent objects were quantified with the following analysis settings: Top-Hat segmentation [radius, 50 µm; threshold, 1 red calibrated unit (RCU); edge split, on; edge sensitivity, −5] and a minimum area filter of 100 µm^2^ to count nuclei but ignore debris.

HFKs were labeled with the Fucci cell cycle reporter system by infection with lentiviruses purchased from the manufacturer (Sartorius), transduced using our lentiviral transduction protocol. Stable Fucci HFK strains were generated via applying the appropriate selection, as described above. Compatible culture plates were scanned in the Incucyte S3 with the ‘Whole Well’ scan type, to ensure all cells in each well were counted. Standard phase-contrast images, red fluorescence images (400 ms acquisition) and green fluorescence images (300 ms acquisition) were collected with the 4× objective. Green fluorescent objects were quantified with the following analysis settings: Top-Hat segmentation [radius, 10 µm; threshold, 1 green calibrated unit (GCU); edge split, on; edge sensitivity, 0], a minimum area filter of 100 µm^2^ to count nuclei and a maximum eccentricity of 0.85. Red fluorescent objects were quantified with the following analysis settings: Top-Hat segmentation [radius, 20 µm; threshold, 1 RCU; edge split, on; edge sensitivity, 0] and a minimum area filter of 100 µm^2^ to count nuclei. Details of reagents can be found in Table S5.

## Supplementary Material

10.1242/joces.260990_sup1 Supplementary information
