# Supplementary material for: Y-27632 acts beyond ROCK inhibition to maintain epidermal stem-like cells in culture
Source: J Cell Sci. 2023 Sep 12;136(17):jcs260990. doi: 10.1242/jcs.260990 (PMC10508688; doi:10.1242/jcs.260990)
Supplement: Supplementary information [file joces-136-260990-s1.pdf]

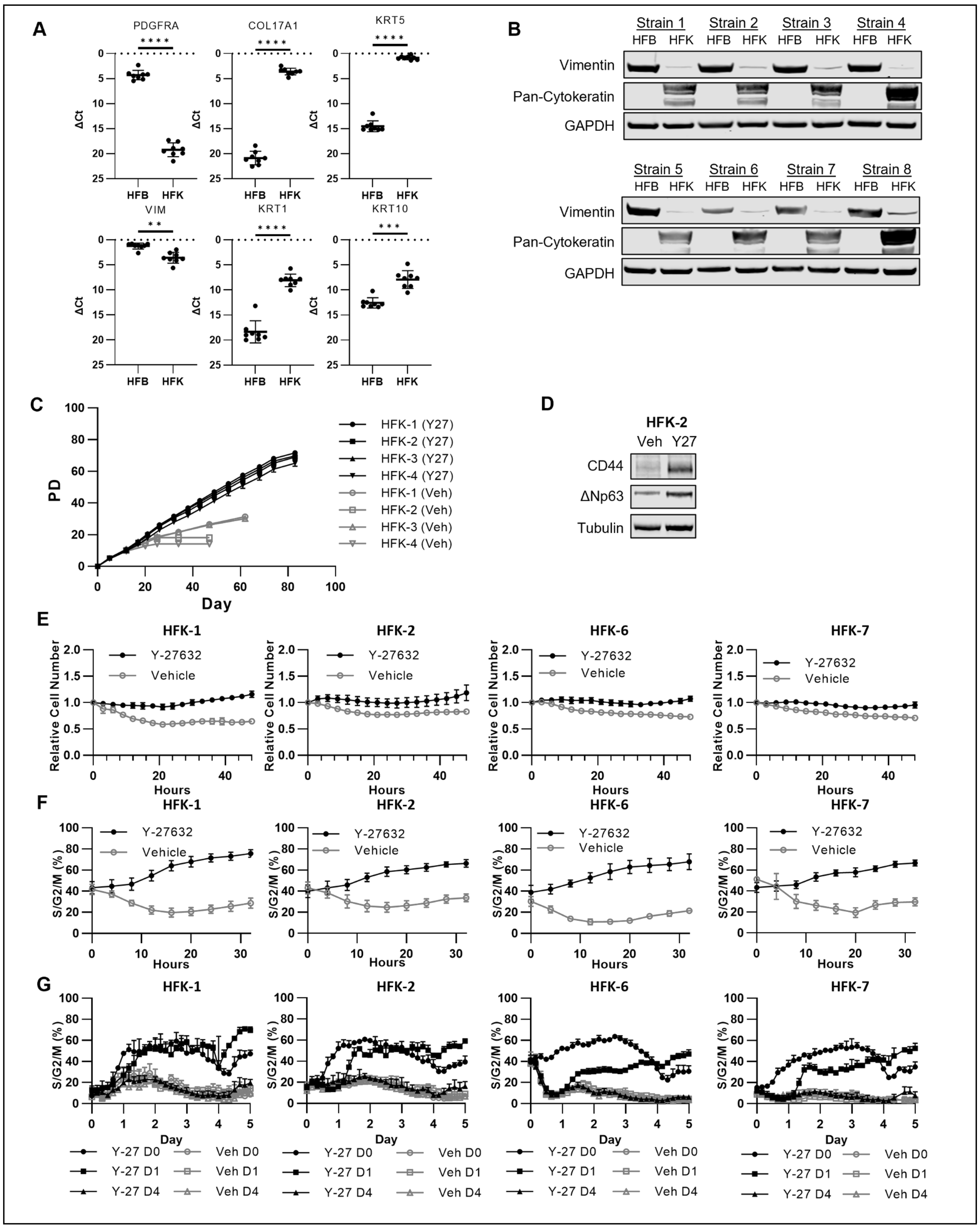

Journal of Cell Science • Supplementary information

Fig. S1. Conditionally reprogrammed human foreskin keratinocyte validation and characterization

A) RT-qPCR was performed with cell lineage-specific markers on cDNA from early passage conditionally reprogrammed human foreskin keratinocyte (HFK) strains or human foreskin fibroblast (HFB) strains isolated from each donor foreskin. Each plotted point represents the mean ΔCt value for each HFK or HFB strain from the eight individual donors used in this study, performed in technical triplicate. Target gene Ct values were normalized to GAPDH as an internal loading control. PDGFRA and VIM served as fibroblast markers while COL17A1, KRT5, KRT1, KRT10 served as keratinocyte markers. Paired t-tests demonstrated significant differences in marker levels for each gene between HFBs and HFKs (****p<0.0001, ***p<0.001, **p<0.01).
B) Western blots on protein lysates from early passage conditionally reprogrammed HFK strains or HFB strains assessed cell lineage-specific marker levels. Vimentin served as a fibroblast marker, while pan-cytokeratin served as a keratinocyte marker.
C) Early passage cultures from four of the eight HFK strains generated in this study were cultured in conditional reprogramming conditions for the first ten population doublings, then cultured in conditioned medium either with 10 µM Y-27632 (Y27) or without Y-27632 (Veh). For all conditions, HFKs were cultured in technical triplicate. Cell counts every five days were used to calculate each culture’s cumulative population doublings (PDs), which were performed in triplicate, averaged across each condition, then plotted.
D) Protein lysates from HFKs cultured five days with (Y27) or without Y-27632 (veh) were harvested to evaluate the expression of the stem cell markers CD44 and ΔNp63. These lysates from an HFK strain were run on Western blots probing for CD44, ΔNp63, and tubulin as a loading control.
E) HFKs from four independent donors were labeled with a nuclear-localized red fluorescent protein (nucRFP) to aid in automated cell counting, then plated either with or without 10 µM Y-27632 following trypsinization in six technical replicates per HFK strain. Viable, adherent HFKs were counted by nucRFP signal every three hours for 48 hours after the HFKs were plated at equal numbers. The number of adherent HFKs at each three-hour time point, relative to the number of initial HFKs plated were plotted, with six technical replicates per strain.
F) Four Fucci-labeled HFK strains were plated with Y-27632, then cultured with or without Y-27632 while RFP and GFP HFKs were counted with an Incucyte automated cell imager every four hours, with six technical replicates per treatment. The fraction of HFKs in S/G2/M over 36 hours for each HFK strain was plotted. Since the S/G2/M and G0/G1 fractions were inverse of one other, we plotted only the fraction of HFKs in S/G2/M. Unpaired t-tests demonstrated a significant difference in S/G2/M fraction at 24 hours (****p<0.0001).
G) Fucci HFK strains were plated with Y-27632, then HFKs were cultured with (Y-27 D0) or without Y-27632 (Veh D0). Additionally, Y-27632 was re-added to HFK cultures after either one day (D1) or four days (D4) without Y-27632, in treatments of technical triplicate. The S/G2/M fraction, for four representative HFK strains was plotted over the course of five days.

Journal of Cell Science • Supplementary information

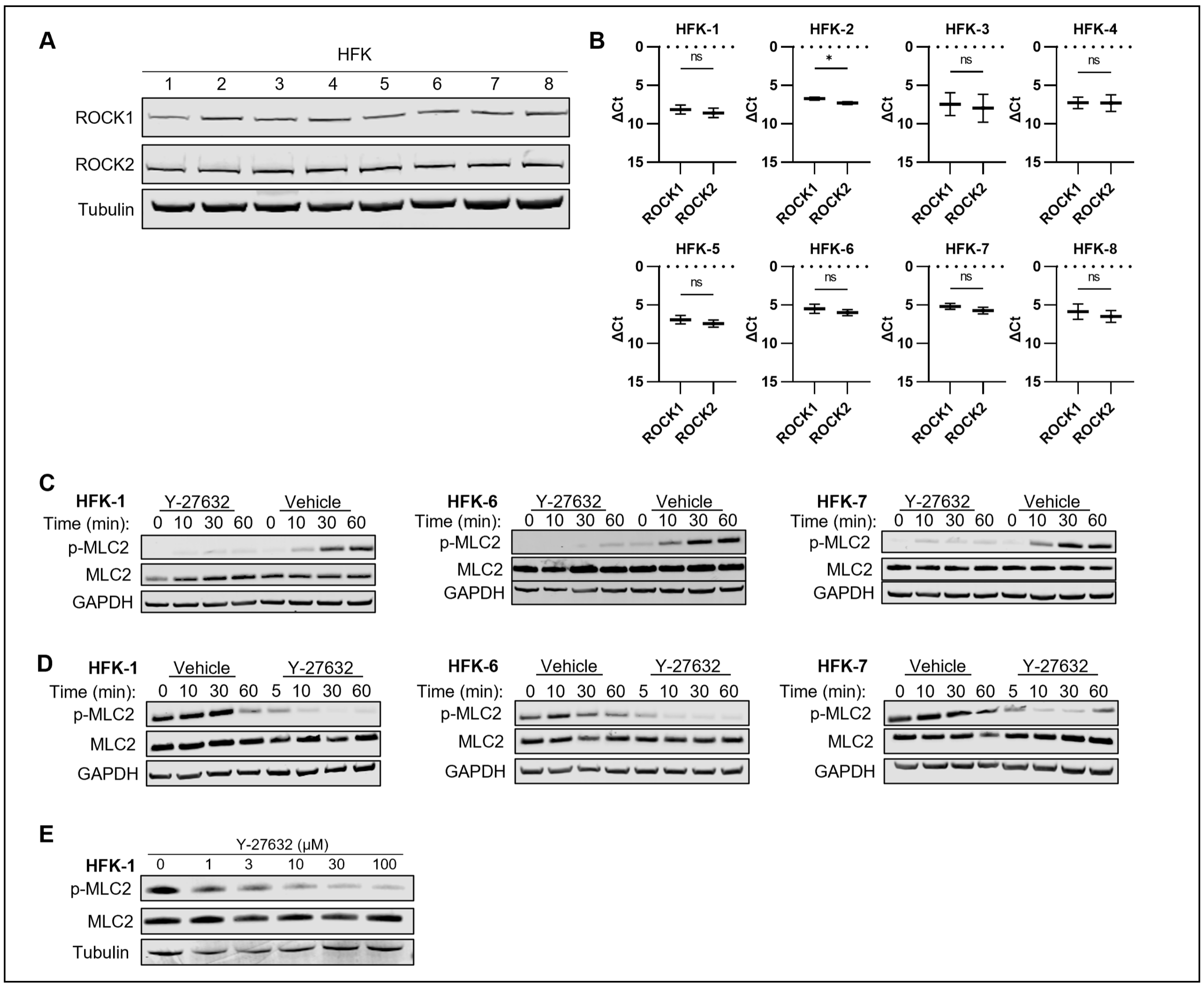

Fig. S2. Conditional Reprogramming limited ROCK activity in HFK cultures

A) Protein was extracted from each conditionally reprogrammed HFK strain isolated in this study (HFK-1 through HFK-8). Protein lysates were run on Western blots to evaluate ROCK1 and ROCK2 expression, with tubulin serving as a loading control.

B) RT-qPCR was performed with ROCK1 and ROCK2 primers on cDNA from each conditionally reprogrammed HFK strain. Each plotted point represents the mean ΔCt value for technical triplicates in each HFK strain. Target gene Ct values were normalized to GAPDH as an internal loading control. Paired t-tests demonstrated similar ROCK isoform mRNA levels within HFK strains (ns $p>0.05$, *$p<0.05$).

C) HFKs were cultured in medium with 10 μM Y-27632, that was removed by PBS wash, and replaced with medium containing vehicle or 10 μM Y-27632. Protein lysates were harvested over the following 60 minutes. To assess ROCK activity, protein lysates were run on Western blots to detect p-MLC2, total MLC2, and GAPDH as a loading control. Replicate Western blots across three HFK strains were performed.

D) Y-27632 was removed with a PBS wash from conditionally reprogrammed HFK cultures, which were cultured with conditioned medium without Y-27632 for one hour to allow ROCK activity to return. Then either vehicle or 10 μM Y-27632 was added to the cultures and protein lysates were harvested over the following 60 minutes. To assess ROCK activity, protein lysates from each of the strains tested were run on Western blots to evaluate p-MLC2, total MLC2, and GAPDH levels. The quantified p-MLC2 signal was normalized to GAPDH and total MLC2 then plotted by time.

E) Another Western blots from a different HFK strain tested Y-27632 concentrations 3-fold and 10-fold higher or lower than the 10 μM Y-27632 used in conditional reprogramming on MLC2 phosphorylation, as described in Fig. 2C.

Journal of Cell Science • Supplementary information

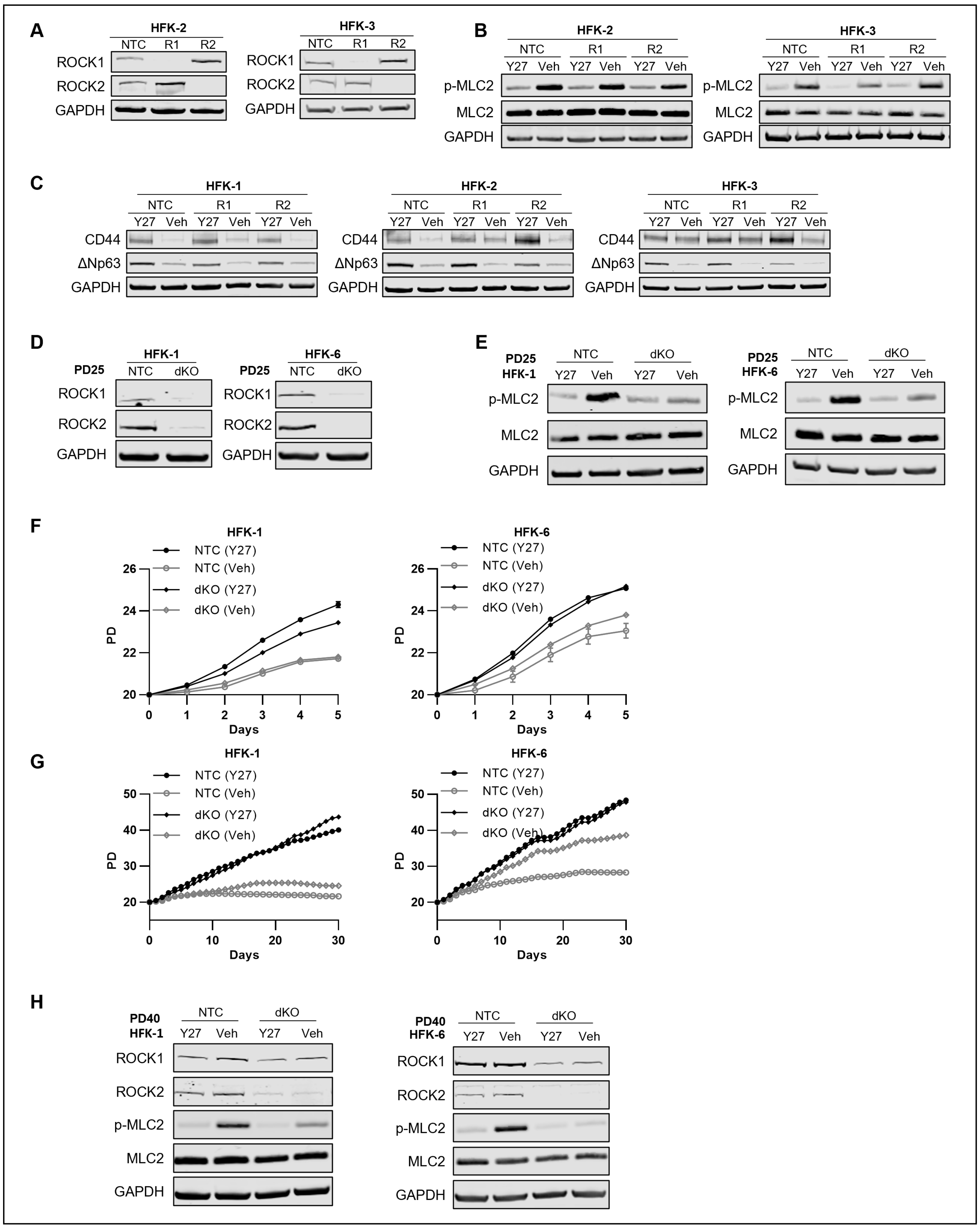

Journal of Cell Science • Supplementary information

Fig. S3. Single or double ROCK isoform deletion did not reprogram HFKs without Y-27632

A) Additional replicates of CRISPR-Cas9-mediated knockouts of either ROCK1 or ROCK2 in other HFK strains are shown. Protein lysates were generated from control (NTC), ROCK1 (R1), and ROCK2 (R2) knockout HFKs and evaluated by Western blot, probing for ROCK1, ROCK2 or GAPDH as a loading control.

B) To evaluate ROCK activity in single ROCK knockout HFKs, protein was extracted from control (NTC), ROCK1 (R1), and ROCK2 (R2) knockouts cultured with or without Y-27632 for one hour. Westerns were probed for MLC2 phosphorylation (p-MLC2) and total MLC2 levels, while GPADH served as a loading control.

C) Single ROCK knockouts from three HFK strains cultured with or without Y-27632 for five days before protein lysates were extracted. These lysates were run on Western blots and probed for levels of the epidermal stem-cell markers CD44 and ΔNp63, while GAPDH as loading control.

D) Replicates of Double ROCK1/ROCK2 CRISPR-Cas9-mediated knockouts were generated in additional HFK strains. Western blots were performed on lysates from control (NTC) HFKs or ROCK double knockout (dKO) HFKs within one passage after CRISPR knockout, probing for ROCK1 and ROCK2, with GAPDH as a loading control.

E) In these additional HFK strains, protein lysates were harvested from control (NTC) or ROCK1/2 knockout (dKO) HFKs within the first passage after CRISPR knockout, cultured either with or without Y-27632 for one hour prior to lysis. Western blots evaluated p-MLC2 and MLC2 total levels with GAPDH as a loading control.

F) Control (NTC) or ROCK double knockout (dKO) HFKs labeled with nucRFP were cultured for 5 days with (Y27) or without Y-27632 (veh), beginning at PD 20. Total cell number was counted daily with an Incucyte automated cell imaging to calculate each culture's cumulative population doublings (PD), plotting the average across three technical triplicates.

G) We extended the experiment with Control (NTC) or ROCK double knockout (dKO) HFKs in Fig. S3F for 30 days with (Y27) or without Y-27632 (veh). Total cell number was counted daily with an Incucyte automated cell imaging to calculate each culture's cumulative population doublings (PD), plotting the average across three technical triplicates.

H) Protein lysates were harvested from control (NTC) or ROCK1/2 knockout (dKO) HFKs from these replicate HFK strains after being cultured for 20 PD in conditional reprogramming conditions, then cultured either with or without Y-27632 for one hour prior to lysis. Western blots evaluated ROCK1, ROCK2, p-MLC2, and MLC2 total levels with GAPDH as a loading control.

Journal of Cell Science • Supplementary information

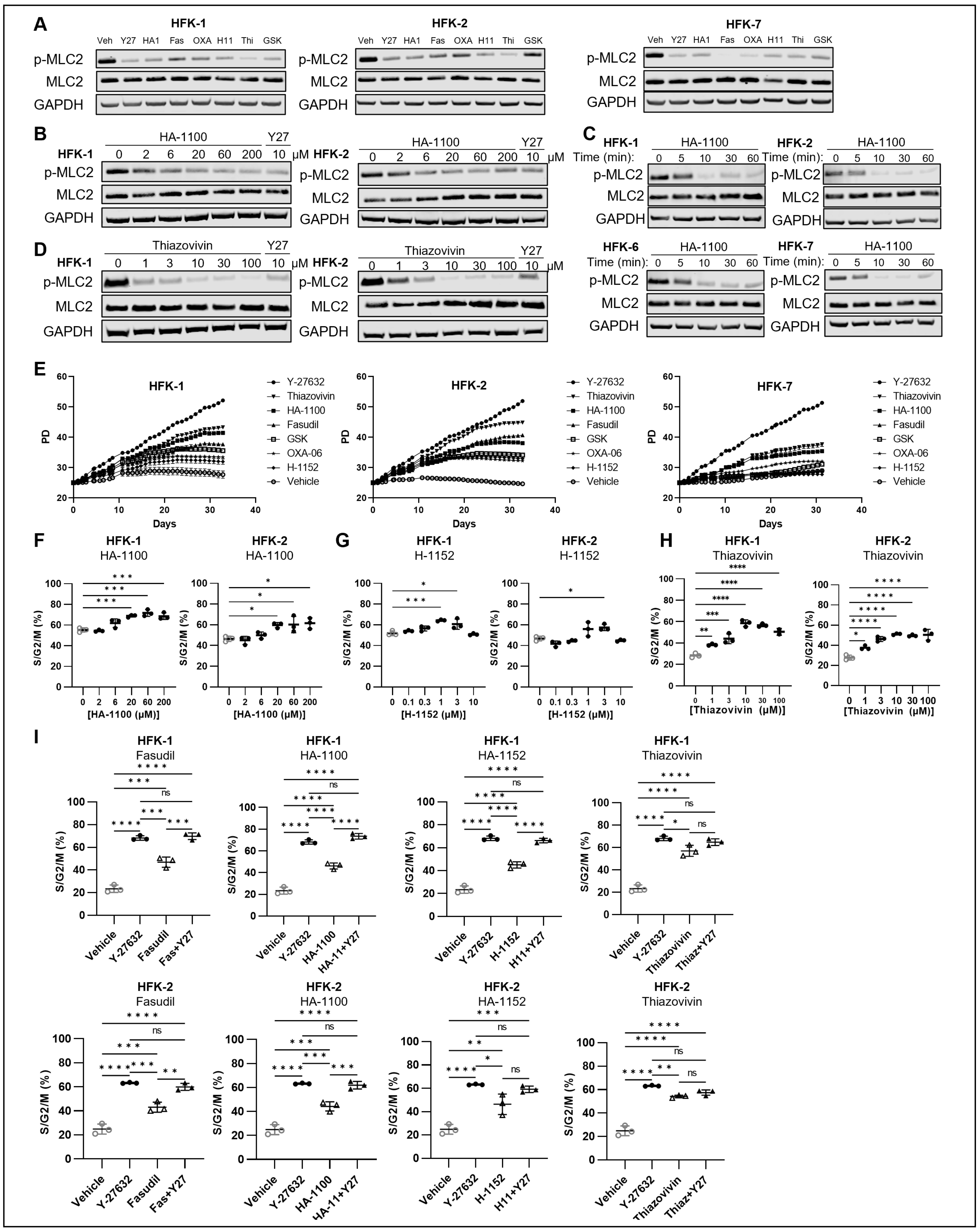

Journal of Cell Science • Supplementary information

Fig. S4. Other ROCK inhibitors differed from Y-27632 in their ability to promote conditional reprogramming
A) Protein lysates were harvested across three additional strains after Y-27632 was removed from HFKs for one hour (Veh) or HFKs were treated with the listed ROCK inhibitor (10 μM Y-27632, 20 μM HA-1100, 20 μM Fasudil, 3 μM OXA-06, 1 μM H-1152, 10 μM Thiazovivin, 100 nM GSK429286) for one hour. Western blots were probed with p-MLC2, total MLC2, and GAPDH to assess ROCK activity.
B) Gradations in HA-1100, 3-fold or 10-fold lower or higher than the optimal dose to decrease MLC2 phosphorylation (20 μM HA-1100), were tested in two HFK strains. Protein lysates were harvested 24 hours following PBS washout and HA-1100 addition. HFKs treated in parallel with 10 μM Y-27632 served as a control. Protein lysates were run on Western blots probed with p-MLC2, total MLC2, and GAPDH to assess ROCK activity.
C) After activating ROCKs by removing Y-27632 for one hour from HFKs, 20 μM HA-1100 was added back and protein lysates were harvested over the following hour. Western blots with these lysates across four HFK strains tested ROCK activity following HA-1100 addition by probing for p-MLC2, total MLC2, and GAPDH. The quantified p-MLC2 signal was normalized to GAPDH and total MLC2 then plotted by time across all four strains.
D) Gradations in Thiazovivin, 3-fold or 10-fold lower or higher than the optimal dose to decrease MLC2 phosphorylation (10 μM Thiazovivin), were tested in two HFK strains. Protein lysates were harvested 1 hour following PBS washout and Thiazovivin addition. HFKs treated in parallel with 10 μM Y-27632 served as a control. Protein lysates were run on Western blots probed with p-MLC2, total MLC2, and GAPDH to assess ROCK activity.
E) Three additional HFK strains were cultured with the other ROCK inhibitors as described in Fig. 4E for 30 days.
F) Gradations in HA-1100, 3-fold or 10-fold lower or higher than the optimal concentration to decrease MLC2 phosphorylation (20 μM HA-1100), were tested in the two Fucci-labeled HFK strains to determine whether increasing gradations of these ROCK inhibitors could increase the fraction of HFKs in S/G2/M. We tracked the distribution of HFKs in G1/G0 and S/G2/M over 48 hours by counting RFP and GFP labeled HFKs with the Incucyte, in technical triplicate, and plotted the average fraction of HFKs in S/G2/M at 24 hours. The mean and deviation across treatments relative to vehicle (0 μM HA-1100) were evaluated via ANOVA (***$p<0.001$, **$p<0.01$, *$p<0.05$).
G) Gradations in H-1152 were tested in the two Fucci labeled HFKs, 3-fold and 10-fold higher or lower than the concentration that maximally decreased p-MLC2 (1 μM H-1152). We tracked the distribution of HFKs in G1/G0 and S/G2/M over 48 hours by counting RFP and GFP labeled HFKs with the Incucyte, in technical triplicate, and plotted the average fraction of HFKs in S/G2/M at 24 hours. The mean and deviation across treatments relative to vehicle (0 μM H-1152) were evaluated via ANOVA (****$p<0.0001$, ***$p<0.001$, **$p<0.01$, *$p<0.05$).
H) Gradations in Thiazovivin were tested in the two Fucci labeled HFKs, 3-fold and 10-fold higher or lower than the concentration that maximally decreased p-MLC2 (10 μM Thiazovivin). We tracked the distribution of HFKs in G1/G0 and S/G2/M over 48 hours by counting RFP and GFP labeled HFKs with the Incucyte, in technical triplicate, and plotted the average fraction of HFKs in S/G2/M at 24 hours. The mean and deviation across treatments relative to vehicle (0 μM Thiazovivin) were evaluated via ANOVA (****$p<0.0001$, ***$p<0.001$, **$p<0.01$, *$p<0.05$).
I) Using two Fucci-labeled HFK strains, Y-27632 was added in combination with other ROCK inhibitors: 20 μM Fasudil (Fas), 20 μM HA-1100 (HA-11), 1 μM H-1152 (H-11), 10 μM Thiazovivin (Thiaz). Conditionally reprogrammed Fucci-labeled HFKs were plated with Y-27632, which was washed out after the HFKs were allowed to adhere overnight. HFKs were cultured in medium containing 10 μM Y-27632 alone, vehicle alone, the ROCK inhibitor listed alone, or the ROCK inhibitor and 10 μM Y-27632 for 36 hours, counting the RFP and GFP labeled HFKs every four hours. We calculated the average fraction of HFKs in S/G2/M at 24 hours, performed in technical triplicate. The mean and deviation across treatments were evaluated via ANOVA ((****$p<0.0001$, ***$p<0.001$, **$p<0.01$, *$p<0.05$, ns $p>0.05$).

Journal of Cell Science • Supplementary information

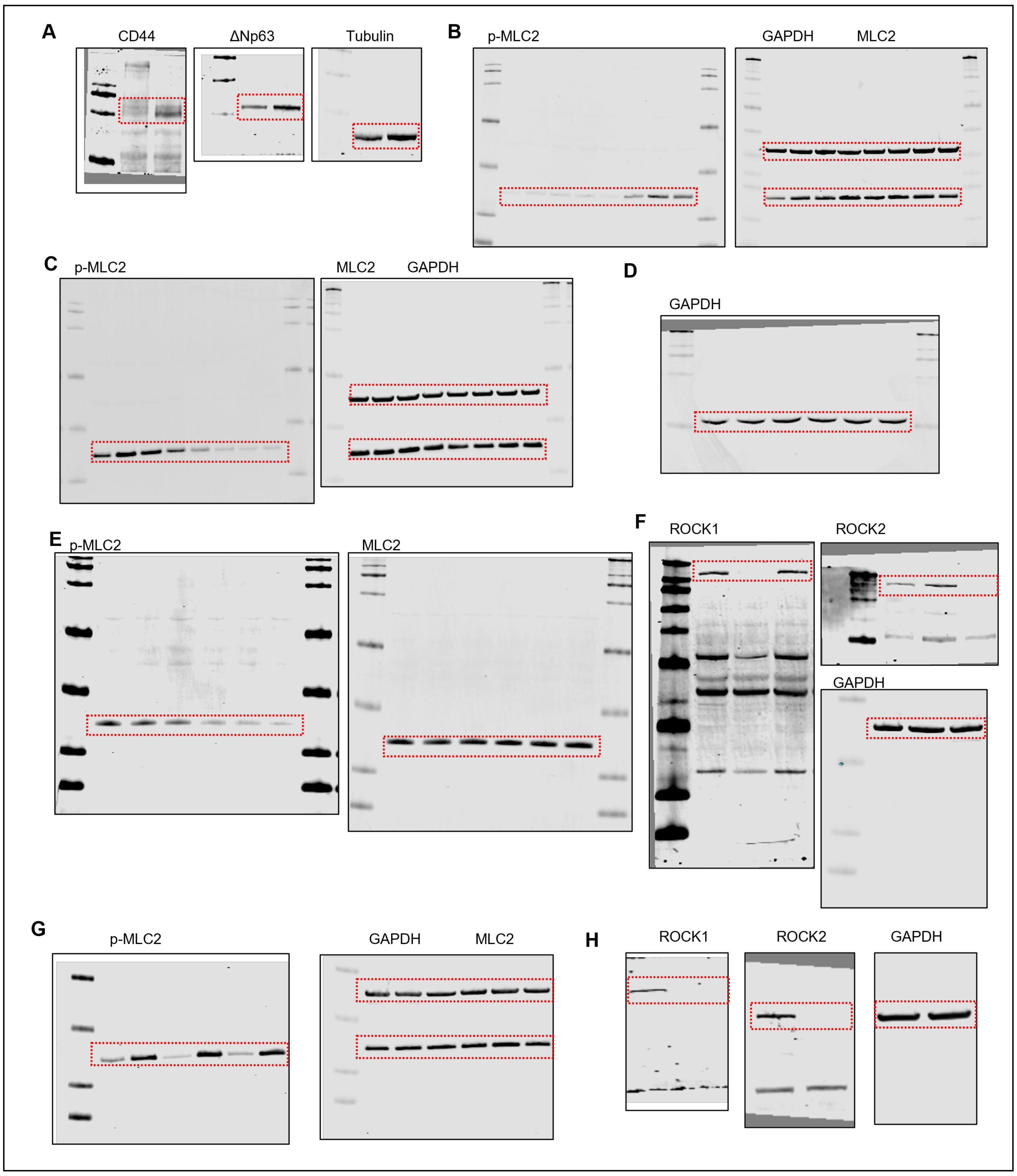

Fig. S5. Western Blot Transparency (part 1)

Uncropped western blots, with a red box to note cropped position, as originally shown in **A)** Figure 1F **B)** Figure 2A **C)** Figure 2B **D-E)** Figure 2C **F)** Figure 3A **G)** Figure 3B **H)** Figure 3D.

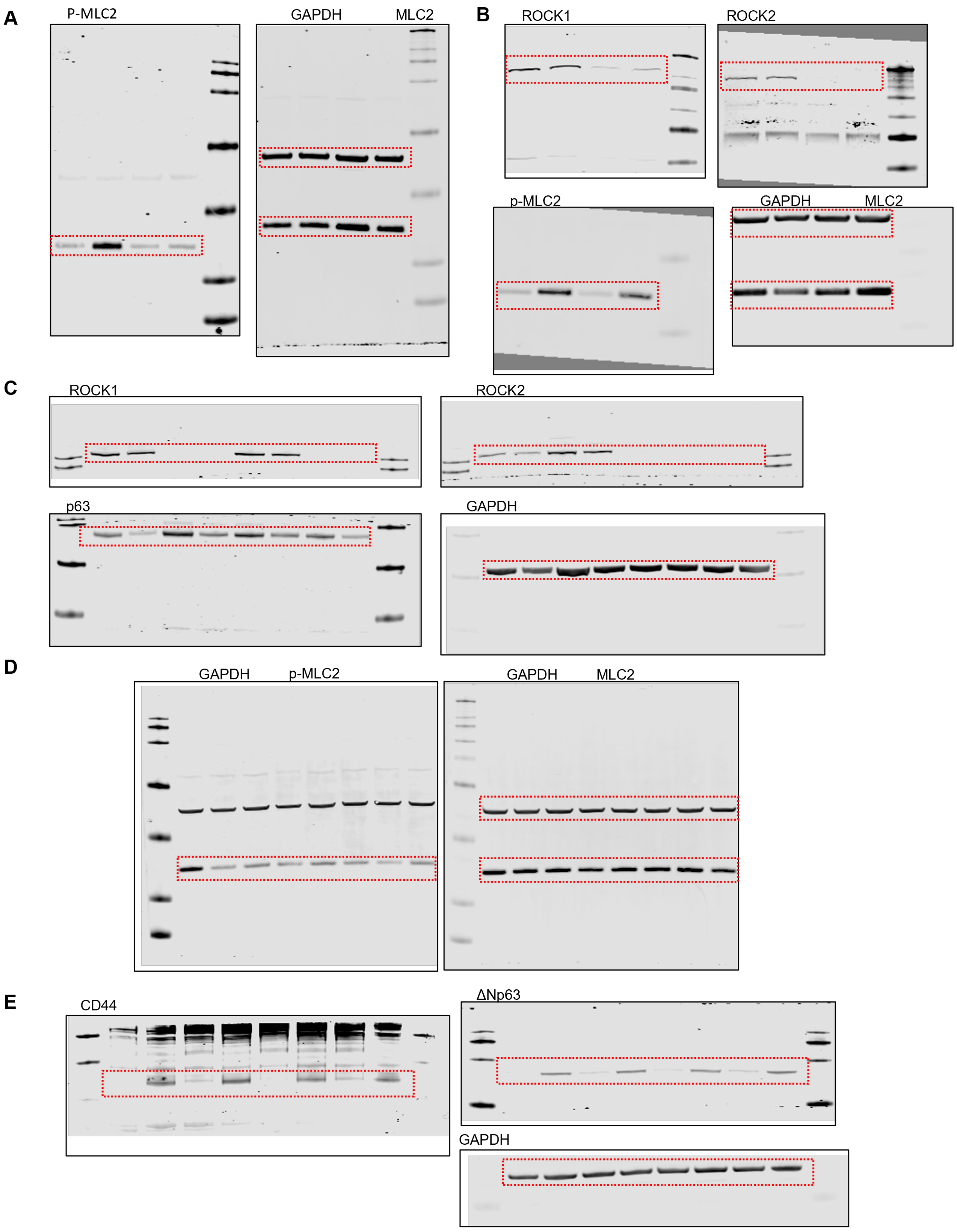

Fig. S6. Western Blot Transparency (part 2)

Uncropped western blots, with a red box to note cropped position, as originally shown in **A)** Figure 3E **B)** Figure 3H **C)** Figure 3I **D)** Figure 4A **E)** Figure 4G

Table S1. ROCK Inhibitor reconstitution

Inhibitor	SOURCE	IDENTIFIER	Solvent	Stock Concentration
Y-27632 dihydrochloride	Enzo	ALX-270-333-M025	H_2O	100 mM
H 1152 dihydrochloride	Tocris	2414	H_2O	10 mM
Fasudil hydrochloride	Tocris	0541	H_2O	100 mM
HA 1100 hydrochloride	Tocris	2415	H_2O	20 mM
GSK 429286	Tocris	3726	DMSO	100 mM
OXA 06 dihydrochloride	Tocris	5182	DMSO	50 mM
Thiazovivin	Tocris	3845	DMSO	100 mM

Table S2. HFK 3F:1D medium components

Component	Final Concentration
Hydrocortisone	0.4 µg/mL
Insulin	5 µg/mL
Cholera Toxin	8.4 ng/mL
EGF	10 ng/mL
Adenine	24 µg/mL
FBS	5% (v/v)
Pen/Strep	1% (v/v)

Table S3. Antibody dilutions for Western Blots

Target	Antibody Name	Dilution	SOURCE	IDENTIFIER
CD44	CD44 (8E2) Mouse mAb #5640	1:500	Cell Signaling Technology	5640
Cytokeratin	Cytokeratin, pan Antibody (AE-1/AE-3)	1:5,000	Novus	NBP2-29429
GAPDH	Anti-GAPDH antibody [6C5] - Loading Control (ab8245)	1:10,000	Abcam	ab8245
MLC2	Myosin Light Chain 2 (D18E2) Rabbit mAb #8505	1:1,000	Cell Signaling Technology	8505
p-MLC2	Phospho-Myosin Light Chain 2 (Thr18/Ser19) Antibody #3674	1:500	Cell Signaling Technology	3674
p63α	p63-α (D2K8X)	1:1,000	Cell Signaling Technology	13109
ROCK1	Recombinant Anti-ROCK1 antibody [EPR638Y]	1:1,000	Abcam	ab134181
ROCK2	ROCK2 Antibody	1:500	Proteintech	66633-1-Ig
Tubulin	β-Tubulin (D2N5G) Rabbit mAb #15115	1:10,000	Cell Signaling Technology	15115
Vimentin	Vimentin (D21H3) XP® Rabbit mAb #5741	1:1,000	Cell Signaling Technology	5741

Journal of Cell Science • Supplementary information

Table S4. RT-qPCR Primer Sequences

Target	Primer Name	Sequence	Source
CD44	F-CD44	CTGCCGCTTTGCAGGTGTA	MGH PrimerBank
	R-CD44	CATTGTGGGCAAGGTGCTATT	
COL17A1	F-COL17A1	TCCACCCGATGGACAGAATTG	MGH PrimerBank
	R-COL17A1	GTAGGTGCCTGACACCGAC	
ΔNp63	F-DNp63	GGGTGATGGAGAGAGCAT	*Cieślar-Pobuda et al 2016*
	R-DNp63	CTGGAAAACAATGCCCAGAC	
GAPDH	F-GAPDH	GAGCCTCAAGATCATCAGCA	*Degen et al 2013*
	R-GAPDH	ACAGTCTTCTGGGTGGCAGT	
KRT1	F-KRT1	AGAGTGGACCAACTGAAGAGT	MGH PrimerBank
	R-KRT1	ATTCTCTGCATTTGTCCGCTT	
KRT5	F-KRT5	TGACCTCCGCAACACCAAG	MGH PrimerBank
	R-KRT5	CAGATTGGCGCACTGTTTCTT	
KRT10	F-KRT10	GGTGGGAGTTATGGAGGCAG	MGH PrimerBank
	R-KRT10	CGAACTTTGTCCAAGTAGGAAGC	
PDGFRA	F-PDGFRA	TGGCAGTACCCCATGTCTGAA	MGH PrimerBank
	R-PDGFRA	CCAAGACCGTCACAAAAAGGC	
ROCK1	F-ROCK1	AACATGCTGCTGGATAAATCTGG	MGH PrimerBank
	R-ROCK1	TGTATCACATCGTACCATGCCT	
ROCK2	F-ROCK2	TCAGAGGTCTACAGATGAAGGC	MGH PrimerBank
	R-ROCK2	CCAGGGGCTATTGGCAAAGG	
Vimentin	F-VIM	AGTCCACTGAGTACCGGAGAC	MGH PrimerBank
	R-VIM	CATTTCACGCATCTGGCGTTC	

Journal of Cell Science • Supplementary information

Table S5. Reagents

EXPERIMENT	REAGENT	SOURCE	IDENTIFIER
Cell Culture and Cell Line Validation Reagents	DMEM, high glucose	Gibco	11965118
	Ham's F-12 Nutrient Mix	Gibco	11765054
	Penicillin-Streptomycin-Glutamine (100X)	Gibco	10378016
	FBS	Sigma	F2442-500ML
	HyClone Bovine Calf Serum	Cytiva	SH30072.03
	Trypsin-EDTA (0.25%), phenol red	Gibco	25200056
	Trypsin-EDTA (0.05%), phenol red	Gibco	25300054
	DMSO (cryopreservation)	Sigma	D2438-50mL
	PBS, pH 7.2	Gibco	20012050
	Trypsin inhibitor from Glycine max (soybean)	Sigma	T9128-1G
	Sodium Pyruvate (100 mM)	Gibco	11360070
	Dispase I	Sigma	D4818
	DNeasy Blood & Tissue Kit	Qiagen	69506
	MycoAlert® PLUS Mycoplasma Detection Kit (100 Tests)	Lonza	LT07-710
Cell Culture related Additives, Inhibitors, and Compounds	Y-27632 . dihydrochloride	Enzo	ALX-270-333-M025
	Hydrocortisone	Sigma	H0888-1G
	Insulin solution from bovine pancreas	Sigma	I0516-5ML
	Cholera Toxin from Vibrio cholerae	Sigma	C8052-.5MG
	EGF	Sigma	E9644-.2MG
	Adenine	Sigma	A8626-1G
	Blasticidin S HCl (10 mg/mL)	Gibco	A1113903
	Hygromycin B (50 mg/mL)	Gibco	10687010
	Puromycin dihydrochloride from Streptomyces alboniger	Sigma	P7255
	Geneticin™ Selective Antibiotic (G418 Sulfate), Powder	Gibco	11811031
	Zeocin	Invivogen	ant-zn-05
	H 1152 dihydrochloride	Tocris	2414
	Fasudil hydrochloride	Tocris	0541
	HA 1100 hydrochloride	Tocris	2415
	GSK 429286	Tocris	3726
	OXA 06 dihydrochloride	Tocris	5182
	Thiazovivin	Tocris	3845
Western Blotting Reagents	Sodium Dodecyl Sulfate (SDS)	Fisher	BP8200500
	DC Protein Assay	Bio-Rad	500-0112
	4x Protein Loading Buffer	Li-Cor	928-40004
	Dithiothreitol	Sigma	43816-10ML
	4%-12% Bis-Tris gels	Invitrogen	NW04120BOX
	Chameleon® Duo Pre-stained Protein Ladder	Li-Cor	928-60000
	Bolt™ MES SDS Running Buffer (20X)	Invitrogen	B0002-02
	Bolt™ MOPS SDS Running Buffer (20X)	Invitrogen	B0001-02
	Tris Base	Fisher	BP152-1
	Glycine	Sigma	G7126
	Methanol	Fisher	A412-4
	PVDF Transfer Membrane, 0.2 µm	Thermo	88520
	Extra Thick Blot Filter Paper, Precut	Bio-Rad	1703966
	10x Tris Buffered Saline (TBS)	Bio-Rad	1706435
	Intercept® (TBS) Blocking Buffer	Li-Cor	927-60001
	TWEEN® 20	Sigma	P2287-500ML
	IRDye 680RD Goat anti-rabbit IgG secondary antibody	Li-Cor	926-68071
	IRDye® 800CW Goat anti-Mouse IgG Secondary Antibody	Li-Cor	926-32210
Lentiviruses and Viral Transductions Reagents	Polyethylenimine, Linear, MW 25000, Transfection Grade (PEI 25K™)	Polysciences	23966
	Opti-MEM	Gibco	31985070
	PEG 8000, Molecular Biology Grade (Polyethylene Glycol 8000)	Promega	V3011
	Sodium Chloride	Fisher	S642-212
	Hexadimethrine bromide	Sigma	H9268
	Incucyte® Nuclight Green Lentivirus (EF1a, Puro)	Sartorius	4476

Journal of Cell Science • Supplementary information

	Incucyte® Nuclight Red Lentivirus (EF1a, Puro)	Sartorius	4476
	Incucyte® Cell Cycle Lentivirus (EF1a, Puro)	Sartorius	4479
RNA isolation & RT-qPCR Reagents	TRIzol™ Reagent	Invitrogen	15596018
	Chloroform	Sigma	C2432
	Isoproanol	Sigma	I9516
	Ethanol	Sigma	E7023
	UltraPure™ DNase/RNase-Free Distilled Water	Invitrogen	10977-015
	RNA Clean & Concentrator-5 (DNase Included)	Zymo Research	R1014
	High-Capacity cDNA Reverse Transcription Kit	Applied Biosystem	4368814
	SsoAdvanced Universal SYBR Green Supermix	Bio-Rad	1725274
Bacterial & Plasmid Preparation Reagents	One Shot™ Stbl3™ Chemically Competent E. coli	Invitrogen	C737303
	LB Broth (Powder) - Lennox	Fisher	BP1427-2
	BACTO™ Agar	BD	214050
	Ampicillin Sodium Salt	Fisher	BP1760-5
	Kanamycin Sulfate	Gibco	11815024
	Spectinomycin dihydrochloride pentahydrate	Sigma	S4014-5G
	QIAprep Spin Miniprep Kit	Qiagen	27106
	NucleoBond® Xtra Midi Plus EF	Macherey-Nagel	740422.50
	Pfu Turbo Hotstart DNA Polymerase	Agilent	600320
	ElectroLigase	NEB	M0369S
	MluI	NEB	R0198S
	BamHI	NEB	R0136S
	BsmBI-v2	NEB	R0739S
	UltraPure™ Agarose	Invitrogen	16500500
	UltraPure™ Ethidium Bromide, 10 mg/mL	Invitrogen	15585011
	QIAEX II Gel Extraction Kit	Qiagen	20021
	LentiCRISPRv2	Addgene	52961
Antibodies	CD44 (8E2) Mouse mAb #5640	Cell Signaling Technology	5640
	Cytokeratin, pan Antibody (AE-1/AE-3)	Novus	NBP2-29429
	Anti-GAPDH antibody [6C5] - Loading Control (ab8245)	Abcam	ab8245
	Myosin Light Chain 2 (D18E2) Rabbit mAb #8505	Cell Signaling Technology	8505
	Phospho-Myosin Light Chain 2 (Thr18/Ser19) Antibody #3674	Cell Signaling Technology	3674
	p63-α (D2K8X)	Cell Signaling Technology	13109
	Recombinant Anti-ROCK1 antibody [EPR638Y]	Abcam	ab134181
	ROCK2 Antibody	Proteintech	66633-1-Ig
	β-Tubulin (D2N5G) Rabbit mAb #15115	Cell Signaling Technology	15115
	Vimentin (D21H3) XP® Rabbit mAb #5741	Cell Signaling Technology	5741
