# Supplementary material for: Y-27632 acts beyond ROCK inhibition to maintain epidermal stem-like cells in culture
Source: J Cell Sci. 2023 Sep 12;136(17):jcs260990. doi: 10.1242/jcs.260990 (PMC10508688; doi:10.1242/jcs.260990)
Supplement: Supplementary file 1 [file joces-136-260990_review_history.pdf]

Y-27632 acts beyond ROCK inhibition to maintain epidermal stem-like cells in culture

Travis A. Witkowski, Bin Li, Jason G. Andersen, Bhavna Kumar, Edmund A. Mroz and James W. Rocco
DOI: 10.1242/jcs.260990

Editor: Daniel Billadeau

Review timeline

Original submission:	17 January 2023
Editorial decision:	21 March 2023
First revision received:	24 May 2023
Editorial decision:	3 July 2023
Second revision received:	21 July 2023
Accepted:	24 July 2023

Original submission

First decision letter

MS ID#: JOCES/2023/260990

MS TITLE: Y-27632 acts beyond ROCK inhibition to maintain epidermal stem-like-cells in culture

AUTHORS: Travis A. Witkowski, Bin Li, Jason G. Andersen, Bhavna Kumar, Edmund A. Mroz, and James W. Rocco
ARTICLE TYPE: Research Article

We have now reached a decision on the above manuscript.

To see the reviewers' reports and a copy of this decision letter, please go to: https://submit-jcs.biologists.org and click on the 'Manuscripts with Decisions' queue in the Author Area. (Corresponding author only has access to reviews.)

As you will see, the reviewers raise a number of substantial criticisms that prevent me from accepting the paper at this stage. They both highlight the lack of mechanistic insight as a major critique of the data. There are several other issues that are also raised by reviewer 1, which would need to be addressed. If you think that you can deal satisfactorily with the criticisms on revision, I would be pleased to see a revised manuscript. We would then return it to the reviewers.

Please ensure that you clearly highlight all changes made in the revised manuscript. Please avoid using 'Tracked changes' in Word files as these are lost in PDF conversion.

I should be grateful if you would also provide a point-by-point response detailing how you have dealt with the points raised by the reviewers in the 'Response to Reviewers' box. Please attend to all of the reviewers' comments. If you do not agree with any of their criticisms or suggestions please explain clearly why this is so.

© 2023. Published by The Company of Biologists under the terms of the Creative Commons Attribution License (https://creativecommons.org/licenses/by/4.0/).

Reviewer 1

Advance summary and potential significance to field

In this manuscript, the ability of Y27632 to influence primary human foreskin keratinocyte (HFK) proliferation and differentiation via inhibition of ROCK kinases has been investigated. Using genetic deletion of ROCK1 and ROCK2 singly or doubly, or a panel of pharmacological ROCK inhibitors, it was determined that the major effect of Y27632 is mediated via ROCK-independent mechanisms. The experiments leading to these conclusions are well designed and mostly convincing.
The manuscript is well organized and clearly written.

Comments for the author

There are important data in Figure 4 that have been downplayed in the manuscript. All four tested ROCK inhibitors have some effect on proliferation differentiation and on long-term growth in Figure 4 that have not been adequately acknowledged. These results actually are consistent with there being some role of ROCK inhibition in the regulation of these processes, even if the effects aren't as great as for Y27632. There may be additional effects derived from Y27632 mediated by ROCK-independent pathways, but the support for there being some contribution from ROCK inhibition should not be overlooked.

Although it is interesting that Y27632 apparently does not mediate its effects on HFK proliferation and differentiation, the results of this study are unlikely to change how this compound is used for the culturing of this primary cell type or how Y27632 is used in other applications. In the final paragraph of the Discussion (lines 313-321), the use of Y27632 in some other applications has been summarized. Although this may help to increase the apparent wide applicability of the present study, it would be more valuable to the research community if there was an indication in the Discussion section of those applications where it is genuinely unclear how Y27632 might be working versus those applications with evidence supporting ROCK inhibition as the primary mechanism of action. For example, the ability of Y27632 to promote the survival of dissociated stem cells (Watanabe et al., Nat Biotechnol. 2007 Jun;25(6):681-6. A ROCK inhibitor permits survival of dissociated human embryonic stem cells.) has clearly been linked to the inhibition of actin-myosin contraction (e.g. Chen et al. Actin-myosin contractility is responsible for the reduced viability of dissociated human embryonic stem cells. Cell Stem Cell. 2010 Aug 6;7(2):240-8.).

A further limitation is that there is no information about the ROCK-independent mechanism of action of Y27632 on HFKs. There is a legitimate argument that characterizing the mechanism of action is beyond the scope of this particular study, but its absence has a significant effect on the novelty and potential impact of the manuscript. Without mechanistic detail, the conclusion that Y27632 acts via a ROCK-independent pathway is interesting but an incremental advance.

As an aside to the content of the manuscript, the job of the reviewer would be made easier if the figures had been numbered, and I suggest that the authors endeavour to include figure numbers in their submissions in the future.

Specific points to consider:
1. Line 34 – "Y-27632 promoted conditional reprogramming by increasing HFK adherence to culture dishes…" Is this what the authors mean to say? Given that the other ROCK inhibitors did increase adherence but not conditional reprogramming, it seems more likely that these are not causally linked. It would be more accurate to say that Y27632 increased HFK adherence and promoted conditional reprogramming.
2. Line 54 – "Conditional reprogramming is so named because this reprogramming into epithelial stem-like-cells is dependent upon regularly adding Y-27632 to cultures. When Y-27632 is removed, epithelial cultures undergo differentiation and stop dividing." It is not clear whether the re-application of Y27632 is due to compound breakdown, is there any evidence for this?
3. Line 168 – "The 10 μM Y-27632 concentration used in conditional reprogramming was the lowest concentration that maximally reduced MLC2 phosphorylation." This statement is not supported by the data in the primary or supplementary figures. Clearly there is additional inhibition of MLC phosphorylation at higher concentrations, making it unclear why this statement was made. It is possible that the higher Y27632 concentrations are inhibiting other MLC kinases.

© 2023. Published by The Company of Biologists under the terms of the Creative Commons Attribution License (https://creativecommons.org/licenses/by/4.0/).

4. Line 215 – "some selection for ROCK1 expression sufficient to restore p-MLC2 levels partially when Y-27632 was removed. (Fig. 3I, Fig. S5E)". This might seem picky, but it would be more accurate to say that the continued passage led to the selection of Y27632-sensitive MLC phosphorylating activity, which likely included a contribution from the increased ROCK1 protein expression. This takes into account the possibility that there are additional kinases that could be sensitive to Y27632 at this concentration that are selected for to overcome the loss of ROCK1 and ROCK2 expression.
5. Line 233 – "This result supports a direct role for ROCKs in the adherence aspect of conditional reprogramming." The effect of ROCK inhibition in promoting adherence is clearly a separate phenomenon from conditional reprogramming. It would be more useful if these two processes were considered as independent.
6. Line 236 – "No other ROCK inhibitor increased the S/G2/M fraction as extensively as Y-27632 did, each providing only a modest S/G2/M fraction increase relative to control. (Fig. 4C)." There are important observations in Figure 4C, which goes counter to the strong conclusions that have been made in the paper. There is a clear and reproducible effect of all four non-Y27632 inhibitors on cell cycle regulation, and on long term proliferation in Figure 4D. In addition, there is some effect of the non-Y27632 inhibitors on the expression of CD44 and deltaNp63. These data actually support the conclusion that there is some role for ROCK inhibition in the regulation of HFK proliferation and differentiation, even if there is an additional contribution from non-ROCK targets in mediating the effect of Y27632. It is very important for the authors to acknowledge these observations.

Reviewer 2

Advance summary and potential significance to field

This manuscript focused on the roles of Y-27632 on the culture of primary human foreskin keratinocyte. The author found that Y-27632 increased HFK adherence to culture dishes, enhanced progression though cell cycle, and promoted stem cell marker expression. It was interesting that the author found ROCK isoform knock out or other ROC inhibitors was not enough to replace Y-27632's role in promoting HFK proliferation. These results indicated the additional roles of Y-2732 except inhibiting ROCKs to sustain the culturing of HFKs. But it is a pity that this issue has been shelved, no in-depth study.

Conditional reprogramming that reprogramming normal epithelial cells into epithelial stem-like-cells is dependent upon regularly adding Y-27632 to cultures, which has been applied clinically. Conditional reprogramming also provides a research model for replicative immortality via maintaining epidermal stem cells. But Y-27632's specific role in conditional reprogramming remains unclear. This manuscript confirmed the facilitation roles of Y-27632 on cell adherence, cell cycle, stem cell survives. It proved the Y-27632 promoted the expression of epidermal stem cell marker in HFKs. These results might be helpful to further clarify the roles of Y-27632 in conditional reprogramming.

Comments for the author

This manuscript found Y-27632 acts beyond ROCK inhibition to maintain epidermal stem-like-cells in culture. But it was just a description of phenotype. The author failed to illuminate the additional roles of Y-27632 except inhibiting ROCKs in promoting HFKs. So the manuscript might not be suitable for publication. Besides, the experimental results section is too tedious, which need to be streamlined by removing some unnecessary logical explanations. The discussion section has the same problem.

In the supplementary information, the author listed experiment repeats of all cell lines, which was not recommended. The pictures of Western blots in Figure S2.E and Figure 1F was exactly the same, which need explain.

© 2023. Published by The Company of Biologists under the terms of the Creative Commons Attribution License (https://creativecommons.org/licenses/by/4.0/).

First revision

Author response to reviewers' comments

We thank the reviewers for a thoughtful evaluation of our manuscript.

The reviewers challenged us to emphasize the significance of the result that Y-27632 induces conditional reprogramming not only by inhibiting ROCKs but also targeting additional unidentified factors(s), without having documented the specific alternate target(s). We are excited the reviewers share the same enthusiasm for identifying the Y-27632's alternate target(s) that we have. We agree this warrants further investigation into what else Y-27632 might target to elicit conditional reprogramming, but also consider this insight alone important to share with the broader scientific community. We aim to share this unexpected result to caution researchers from equating Y-27632's effects with activity upon the ROCKs in its uses for conditional reprogramming and reprogramming for other cell types, as is now often done in the literature.

The reviewers also asked us to discuss the finding that the ROCKs may still contribute some effects towards conditional reprogramming, which we addressed with additional revisions to the text. Some of Y-27632's effects upon HFK cultures, like promoting adhesion, appear to be direct ROCK effects. As our experiments could not rule out that promoting cellular adhesion may contribute to conditional reprogramming, we did not separate out these effects. The Western blot added to Figure 3I emphasizes that the increase in epidermal stem-cell markers required Y-27632 even when ROCKs were deleted. These results showed features in addition to ROCK inhibition from Y-27632 contribute towards epidermal stem cell renewal but could not determine whether the increase in stem-cell markers with Y-27632 was entirely ROCK- independent. As such we have endeavored to emphasize this in the text.

To address other more specific concerns from review, please see below and the attached document containing our detailed responses.

Reviewer 1 Advance Summary and Potential Significance to Field...
In this manuscript, the ability of Y27632 to influence primary human foreskin keratinocyte (HFK) proliferation and differentiation via inhibition of ROCK kinases has been investigated. Using genetic deletion of ROCK1 and ROCK2 singly or doubly, or a panel of pharmacological ROCK inhibitors, it was determined that the major effect of Y27632 is mediated via ROCK- independent mechanisms. The experiments leading to these conclusions are well designed and mostly convincing. The manuscript is well organized and clearly written.

Reviewer 1 Comments for the Author...
There are important data in Figure 4 that have been downplayed in the manuscript. All four tested ROCK inhibitors have some effect on proliferation, differentiation and on long-term growth in Figure 4 that have not been adequately acknowledged. These results actually are consistent with there being some role of ROCK inhibition in the regulation of these processes, even if the effects aren't as great as for Y27632. There may be additional effects derived from Y27632 mediated by ROCK-independent pathways, but the support for there being some contribution from ROCK inhibition should not be overlooked.

In our excitement to share our finding that activity beyond ROCK inhibition is required for conditional reprogramming, we may not have clearly conveyed that the results indicate the ROCKs likely still play some role in these processes. We thank the reviewer for bringing this to our attention and have addressed this throughout the manuscript as follows: lines 27, 42-44, 97-98, 265-267, 288-291, 300-301.

Although it is interesting that Y27632 apparently does not mediate its effects on HFK proliferation and differentiation, the results of this study are unlikely to change how this compound is used for the culturing of this primary cell type, or how Y27632 is used in other applications. In the final paragraph of the Discussion (lines 313-321), the use of Y27632 in some other applications has been summarized. Although this may help to increase the apparent wide applicability of the present

© 2023. Published by The Company of Biologists under the terms of the Creative Commons Attribution License (https://creativecommons.org/licenses/by/4.0/).

study, it would be more valuable to the research community if there was an indication in the Discussion section of those applications where it is genuinely unclear how Y27632 might be working versus those applications with evidence supporting ROCK inhibition as the primary mechanism of action. For example, the ability of Y27632 to promote the survival of dissociated stem cells (Watanabe et al., Nat Biotechnol. 2007 Jun;25(6):681-6. A ROCK inhibitor permits survival of dissociated human embryonic stem cells.) has clearly been linked to the inhibition of actin-myosin contraction (e.g. Chen et al. Actin-myosin contractility is responsible for the reduced viability of dissociated human embryonic stem cells. Cell Stem Cell. 2010 Aug 6;7(2):240-8.). A further limitation is that there is no information about the ROCK-independent mechanism of action of Y27632 on HFKs. There is a legitimate argument that characterizing the mechanism of action is beyond the scope of this particular study, but its absence has a significant effect on the novelty and potential impact of the manuscript. Without mechanistic detail, the conclusion that Y27632 acts via a ROCK-independent pathway is interesting but an incremental advance.

We investigated the role of Y-27632 in conditional reprogramming to study how the ROCKs controlled epidermal stem-cell renewal. We thank the reviewer for highlighting our inadequacies in conveying the significance of this work, which we have addressed by adding the following text: lines 35, 42-44, 57-59, 97-98. As ROCKs have been shown to directly play a role in cell adhesion for other cell types, like iPSCs, we emphasized this ROCK-specific effect from Y-27632 with changes to lines 120-121 and 326-328. We highlighted the potential ROCK-independent role in stem-cell control in 331-333.

As an aside to the content of the manuscript, the job of the reviewer would be made easier if the figures had been numbered, and I suggest that the authors endeavour to include figure numbers in their submissions in the future.

We apologize for the inconvenience and will address this in future submissions.

Specific points to consider:

1. Line 34 – “Y-27632 promoted conditional reprogramming by increasing HFK adherence to culture dishes…” Is this what the authors mean to say? Given that the other ROCK inhibitors did increase adherence but not conditional reprogramming, it seems more likely that these are not causally linked. It would be more accurate to say that Y27632 increased HFK adherence and promoted conditional reprogramming.

We have incorporated this comment into the new version in lines 35-36.

2. Line 54 – “Conditional reprogramming is so named because this reprogramming into epithelial stem-like-cells is dependent upon regularly adding Y-27632 to cultures. When Y-27632 is removed, epithelial cultures undergo differentiation and stop dividing.” It is not clear whether the re-application of Y27632 is due to compound breakdown, is there any evidence for this?

We change the conditioned medium and add fresh Y-27632 to HFK cultures every 48 hours. Experiments from other labs suggest that Y-27632 remains stable in medium used to culture pluripotent stem cells over this timeframe. *So et al 2020* (10.1371/journal.pone.0233057).

3. Line 168 – “The 10 μM Y-27632 concentration used in conditional reprogramming was the lowest concentration that maximally reduced MLC2 phosphorylation.” This statement is not supported by the data in the primary or supplementary figures. Clearly there is additional inhibition of MLC phosphorylation at higher concentrations, making it unclear why this statement was made. It is possible that the higher Y27632 concentrations are inhibiting other MLC kinases.

We have incorporated this comment into the new version in lines 174-176.

4. Line 215 – “some selection for ROCK1 expression sufficient to restore p-MLC2 levels partially when Y-27632 was removed. (Fig. 3I, Fig. S5E)”. This might seem picky, but it would be more accurate to say that the continued passage led to the selection of Y27632- sensitive MLC phosphorylating activity, which likely included a contribution from the increased ROCK1 protein expression. This takes into account the possibility that there are additional kinases that could be sensitive to Y27632 at this concentration that are selected for to overcome the loss of ROCK1 and

© 2023. Published by The Company of Biologists under the terms of the Creative Commons Attribution License (https://creativecommons.org/licenses/by/4.0/).

ROCK2 expression.

We have incorporated this comment into the new version in lines 221-223.

5. Line 233 – “This result supports a direct role for ROCKs in the adherence aspect of conditional reprogramming.” The effect of ROCK inhibition in promoting adherence is clearly a separate phenomenon from conditional reprogramming. It would be more useful if these two processes were considered as independent.

We agree that Y-27632’s ability to promote HFK adhesion does not directly promote conditional reprogramming. However, we have not separated these two effects as entirely independent of one another as we did not directly test the hypothesis that conditional reprogramming does not require the adhesion effect from Y-27632.

6. Line 236 – “No other ROCK inhibitor increased the S/G2/M fraction as extensively as Y-27632 did, each providing only a modest S/G2/M fraction increase relative to control. (Fig. 4C).” There are important observations in Figure 4C, which goes counter to the strong conclusions that have been made in the paper. There is a clear and reproducible effect of all four non-Y27632 inhibitors on cell cycle regulation, and on long term proliferation in Figure 4D. In addition, there is some effect of the non-Y27632 inhibitors on the expression of CD44 and deltaNp63. These data actually support the conclusion that there is some role for ROCK inhibition in the regulation of HFK proliferation and differentiation, even if there is an additional contribution from non-ROCK targets in mediating the effect of Y27632. It is very important for the authors to acknowledge these observations.

We have addressed this point with the changes mentioned above (lines 27, 42-44, 97-98, 265-267, 288-291, 300-301)

Reviewer 2 Advance Summary and Potential Significance to Field...

This manuscript focused on the roles of Y-27632 on the culture of primary human foreskin keratinocyte.

The author found that Y-27632 increased HFK adherence to culture dishes, enhanced progression though cell cycle, and promoted stem cell marker expression. It was interesting that the author found ROCK isoform knock out or other ROC inhibitors was not enough to replace Y-27632’s role in promoting HFK proliferation. These results indicated the additional roles of Y-2732 except inhibiting ROCKs to sustain the culturing of HFKs. But it is a pity that this issue has been shelved, no in-depth study.

Conditional reprogramming that reprogramming normal epithelial cells into epithelial stem- like-cells is dependent upon regularly adding Y-27632 to cultures, which has been applied clinically. Conditional reprogramming also provides a research model for replicative immortality via maintaining epidermal stem cells. But Y-27632’s specific role in conditional reprogramming remains unclear. This manuscript confirmed the facilitation roles of Y-27632 on cell adherence, cell cycle, stem cell survives. It proved the Y-27632 promoted the expression of epidermal stem cell marker in HFKs. These results might be helpful to further clarify the roles of Y-27632 in conditional reprogramming.

Reviewer 2 Comments for the Author...

This manuscript found Y-27632 acts beyond ROCK inhibition to maintain epidermal stem- like-cells in culture. But it was just a description of phenotype. The author failed to illuminate the additional roles of Y-27632 except inhibiting ROCKs in promoting HFKs. So the manuscript might not be suitable for publication. Besides, the experimental results section is too tedious, which need to be streamlined by removing some unnecessary logical explanations. The discussion section has the same problem.

© 2023. Published by The Company of Biologists under the terms of the Creative Commons Attribution License (https://creativecommons.org/licenses/by/4.0/).

We thank the reviewer for their enthusiasm that we share to identify what else Y-27632 acts upon to elicit conditional reprogramming. We wanted to share these unexpected findings that Y-27632 requires additional activity with the scientific community, as we investigate this further.

In the supplementary information, the author listed experiment repeats of all cell lines, which was not recommended. The pictures of Western blots in Figure S2.E and Figure 1F was exactly the same, which need explain.

We thank the reviewer for their attentiveness to identify that we inadvertently copied the same western blot in the supplement as in the main figures. We have addressed this issue. We included replicates of our HFK strains to emphasize the reproducibility of our results.

Second decision letter

MS ID#: JOCES/2023/260990

MS TITLE: Y-27632 acts beyond ROCK inhibition to maintain epidermal stem-like-cells in culture

AUTHORS: Travis A Witkowski, Bin Li, Jason G Andersen, Bhavna Kumar, Edmund A Mroz, and James W Rocco
ARTICLE TYPE: Research Article

Could you please address point 2 of reviewer 2. There is no need to perform single cell cloning of DKO ROCK1/2 KO cells.

Please ensure that you clearly highlight all changes made in the revised manuscript. Please avoid using 'Tracked changes' in Word files as these are lost in PDF conversion.

I should be grateful if you would also provide a point-by-point response detailing how you have dealt with the points raised by the reviewers in the 'Response to Reviewers' box. Please attend to all of the reviewers' comments. If you do not agree with any of their criticisms or suggestions please explain clearly why this is so.

Reviewer 1

Advance summary and potential significance to field

I am satisfied with the revisions made in response to my original comments.

Comments for the author

I am satisfied with the revisions made in response to my original comments.

Reviewer 2

Advance summary and potential significance to field

In this manuscript, ROCK knock out or other ROCK inhibitors were found not enough to replace Y-27632's role in promoting condition reprogramming. These results indicated the additional roles of Y-2732 except inhibiting ROCKs to sustain the culturing of HFKs. This discovery of Y27632's role in conditional reprogramming retains its novelty. These results might be helpful to further clarify the roles of Y-27632 in conditional reprogramming.

© 2023. Published by The Company of Biologists under the terms of the Creative Commons Attribution License (https://creativecommons.org/licenses/by/4.0/).

Comments for the author

The authors made it clear that the conclusion Y27632 promotes conditional reprogramming beyond inhibiting ROCK has certain positive significance. The conclusion was mainly derived from that ROCK knockout or compound substitution failed to replace Y27632 promoting conditional reprogramming. But in the ROCK knockout parts, knockout cells were heterogenous and might not suitable for long-term culture. In the revised manuscript, wild-type cells were found to restore in long-term culture without Y27632. Therefore, relevant experiments are suggested to be repeated with more reliable experimental materials. In compound substitution parts, some compounds such as Thiazovivin show a slightly weaker effect similar to Y27632. But the effect of gradient concentration was not explored, which affects reliability of the conclusion ROCK inhibitors differed from Y-27632 on promoting conditional reprogramming. Detailed comments are as followed.

1) Cells used for CRISPR ROCK deletion were achieved after transduction with CRISPR-containing lentivirus. That might give rise to some unexpected wild type cells mixed in these knock-out cells. In Figure S3H, the ROCK1 was still expressed in the dKO cells, which prove the worry. Therefore it is recommended to generate ROCK1 and ROCK2 knock out cell lines by single cell cloning to explore Y27632's effect on cell propagation.

2) In Figure 4G, Thiazovivin promoted the weak expression of CD 44 and δNp63, showing the possibility that Thiazovivin renews epidermal stem cells. In addition, among all alternatives, Thiazovivin exhibited the best promotion of long-term cell culture, and the ratio of S/G2/M cells was also higher compared to other small molecules. Therefore, it is recommended to explore the substitution effect of Thiazovivin in high concentration. Otherwise, the relevant experimental results could not show that other ROCK inhibitors are really different from Y27632 in their ability to promote conditional reprogramming.

Second revision

Author response to reviewers' comments
From the Editor
Could you please address point 2 of reviewer 2. There is no need to perform single cell cloning of DKO ROCK1/2 KO cells.

Please ensure that you clearly highlight all changes made in the revised manuscript. Please avoid using 'Tracked changes' in Word files as these are lost in PDF conversion.

I should be grateful if you would also provide a point-by-point response detailing how you have dealt with the points raised by the reviewers in the 'Response to Reviewers' box. Please attend to all of the reviewers' comments. If you do not agree with any of their criticisms or suggestions please explain clearly why this is so.

We thank you for your atention to our manuscript, and the reviewers for thoughtiul critiques of the revised manuscript. As Reviewer 1 raised no new points and you have indicated that single cell cloning is not required, we respond specitically to point 2 of Reviewer 2.

Reviewer 1 Advance Summary and Potential Significance to Field:
I am satisfied with the revisions made in response to my original comments.

Reviewer 1 Comments for the Author:
I am satistied with the revisions made in response to my original comments.

OK, thank you.

Reviewer 2 Advance Summary and Potential Significance to Field:
In this manuscript, ROCK knock out or other ROCK inhibitors were found not enough to replace Y-27632's role in promoting condition reprogramming. These results indicated the additional roles of

© 2023. Published by The Company of Biologists under the terms of the Creative Commons Attribution License (https://creativecommons.org/licenses/by/4.0/).

Y-2732 except inhibiting ROCKs to sustain the culturing of HFKs. This discovery of Y27632's role in conditional reprogramming retains its novelty. These results might be helpful to further clarify the roles of Y-27632 in conditional reprogramming.

<u>**Reviewer 2** Comments for the Author:</u>
The authors made it clear that the conclusion Y27632 promotes conditional reprogramming beyond inhibiting ROCK has certain positive signiticance. The conclusion was mainly derived from that ROCK knockout or compound substitution failed to replace Y27632 promoting conditional reprogramming. But in the ROCK knockout parts, knockout cells were heterogenous and might not suitable for long-term culture. In the revised manuscript, wild-type cells were found to restore in long-term culture without Y27632. Therefore, relevant experiments are suggested to be repeated with more reliable experimental materials. In compound substitution parts, some compounds such as Thiazovivin show a slightly weaker etiect similar to Y27632. But the etiect of gradient concentration was not explored, which atiects reliability of the conclusion ROCK inhibitors ditiered from Y-27632 on promoting conditional reprogramming. Detailed comments are as followed.

1) Cells used for CRISPR ROCK deletion were achieved atier transduction with CRISPR-containing lentivirus. That might give rise to some unexpected wild type cells mixed in these knock-out cells. In Figure S3H, the ROCK1 was still expressed in the dKO cells, which prove the worry. Therefore it is recommended to generate ROCK1 and ROCK2 knock out cell lines by single cell cloning to explore Y27632's etiect on cell propagation

Please see above.
2) In Figure 4G, Thiazovivin promoted the weak expression of CD 44 and tiNp63, showing the possibility that Thiazovivin renews epidermal stem cells. In addition, among all alternatives, Thiazovivin exhibited the best promotion of long-term cell culture, and the ratio of S/G2/M cells was also higher compared to other small molecules. Therefore, it is recommended to explore the substitution etiect of Thiazovivin in high concentration. Otherwise, the relevant experimental results could not show that other ROCK inhibitors are really ditierent from Y27632 in their ability to promote conditional reprogramming.

As a single agent, Thaizovivin showed maximal etiects on MLC2 phosphorylation, expression of stem-cell markers CD44 and tiNp63, and proliferation (as assessed by FUCCI) at the concentration of 10 tiM reported in the manuscript. Please see the data added to Figure S4 D and H, where we showed higher Thiazovivin concentrations beyond 10 tiM did not further alter ROCK activity or HFK proliferation (asseded by FUCCI). As discussed in lines 298 to 305 of the manuscript, at this optimal concentration the etiects of Thaizovivin were less than those of 10 tiM Y-27632. Furthermore, adding 10 tiM Y-27632 to 10 tiM Thiazovivin led to even more rapid proliferation and expression of stem-cell markers, despite having no additional inhibition of MLC2 phosphorylation. This further supports the idea that Y27632 has etiects beyond inhibition of ROCKs to promote conditional reprogramming.

<u>Third decision letter</u>

MS ID#: JOCES/2023/260990

MS TITLE: Y-27632 acts beyond ROCK inhibition to maintain epidermal stem-like-cells in culture

AUTHORS: Travis A Witkowski, Bin Li, Jason G Andersen, Bhavna Kumar, Edmund A Mroz, and James W Rocco
ARTICLE TYPE: Research Article

I am happy to tell you that your manuscript has been accepted for publication in Journal of Cell Science, pending standard ethics checks.

© 2023. Published by The Company of Biologists under the terms of the Creative Commons Attribution License (https://creativecommons.org/licenses/by/4.0/).
